# Development and Initial Validation of an Acute Readiness Monitoring Scale in Military Personnel

**DOI:** 10.3389/fpsyg.2021.738609

**Published:** 2021-11-18

**Authors:** Richard James Keegan, Andrew Flood, Theo Niyonsenga, Marijke Welvaert, Ben Rattray, Mustafa Sarkar, Lee Melberzs, David Crone

**Affiliations:** ^1^Research Institute for Sport and Exercise, Faculty of Health, University of Canberra, Canberra, ACT, Australia; ^2^Faculty of Health, University of Canberra, Canberra, ACT, Australia; ^3^Health Research Institute, University of Canberra, Canberra, ACT, Australia; ^4^Australian National University, Canberra, ACT, Australia; ^5^School of Science and Technology, Nottingham Trent University, Nottingham, United Kingdom; ^6^Australian Army, Adelaide, SA, Australia; ^7^Department of Defence, Australian Government, Edinburgh, SA, Australia

**Keywords:** job performance/performance measurement, job design/work characteristics/empowerment, psychometrics/measurement, mood, fatigue, subjective ratings, monitoring

## Abstract

Personnel in many professions must remain “ready” to perform diverse activities. Managing individual and collective capability is a common concern for leadership and decision makers. Typical existing approaches for monitoring readiness involve keeping detailed records of training, health and equipment maintenance, or – less commonly – data from wearable devices that can be difficult to interpret as well as raising privacy concerns. A widely applicable, simple psychometric measure of perceived readiness would be invaluable in generating rapid evaluations of current capability directly from personnel. To develop this measure, we conducted exploratory factor analysis and confirmatory factor analysis with a sample of 770 Australian military personnel. The 32-item Acute Readiness Monitoring Scale (ARMS) demonstrated good model fit, and comprised nine factors: overall readiness; physical readiness; physical fatigue; cognitive readiness; cognitive fatigue; threat-challenge (i.e., emotional/coping) readiness; skills-and-training readiness; group-team readiness, and equipment readiness. Readiness factors were negatively correlated with recent stress, current negative affect and distress, and positively correlated with resilience, wellbeing, current positive affect and a supervisor’s rating of solider readiness. The development of the ARMS facilitates a range of new research opportunities: enabling quick, simple and easily interpreted assessment of individual and group readiness.

## Introduction

Maintaining capabilities spanning physical, cognitive, emotional, skill and team domains are common to many professions, for example: construction (Yip and Rowlinson, 2006; Nahrgang et al., 2011); nursing (Kuiper and Pesut, 2004); medicine/surgery (Elfering et al., 2017); sales/marketing (McFarland et al., 2016), and many more (Bakker and Demerouti, 2007). Research in occupational psychology offers frameworks such as the Job Demands–Resources (JD-R) model (Bakker and Demerouti, 2007), which specifies that the balancing of these capabilities against job demands predicts: job burnout (e.g., Demerouti et al., 2001; Bakker et al., 2005), organizational commitment, work enjoyment (Bakker et al., 2010), connectedness (Lewig et al., 2007), and work engagement (Bakker et al., 2007). At present, the majority of this balancing – through careful management, planning, training and recruitment – remains a challenging, time-demanding and slow (or infrequent) task, with diverse approaches and methods deployed in different contexts (Chuang et al., 2016; Der-Martirosian et al., 2017; Shah et al., 2017; Sharma et al., 2018). Against this backdrop, offering a brief, psychometrically validated, easily interpreted and widely applicable assessment of acute performance readiness may substantially improve the performance planning of personnel and their managers.

In the military, just as in other occupations, individuals and groups are developed, supported and assessed in relation to their integrated capabilities, encompassing the physical, cognitive, and psychological, as well as role-specific skills, team functioning, and equipment (Peterson et al., 2011; Reivich et al., 2011; Training and Doctrine Command, 2011). Indeed, military personnel around the world perform important roles extending beyond direct combat, to include: remote operations; peacekeeping (Bester and Stanz, 2007); protecting key assets (borders, heritage sites, endangered species); cyber activities; emergency responding (e.g., floods, bushfires, earthquakes); and collaborating with other nations (Rollins, 2001; Faber et al., 2008; Apiecionek et al., 2012; Roy and Lopez, 2013). In this way, the diversity of military roles reflects many professional and occupational settings. This diversity comprises a complex array of duties, priorities, and constraints: requiring individuals to be highly capable, strategically adaptive, and prepared to the highest standards (Rutherford, 2013). Accordingly, personnel must remain *ready* to perform their job role, across a wide array of capabilities, often for prolonged periods of time.

### Readiness Profiles as Indicative of Resilience

As well as seeking to facilitate improved management of role readiness in acute timeframes, we also sought to respond to recent critiques of the concept of resilience – which has been adopted in workplaces around the world (Robertson et al., 2015; Crane, 2017). Current research has tended to focus on stable individual attributes that predict positive adaptations to adversity (“stable protective or mitigating personal factors” – e.g., Rutter, 1987; Connor and Davidson, 2003). This focus has constrained the ability to also examine resilience’s theoretical emphasis on the acute interaction of person-in-environment and the dynamic, interactive nature of resilience (Windle et al., 2011; Pangallo et al., 2015). Further, most current research in resilience characterizes the “positive adaptations” as exclusively referring to mental health or subjective wellbeing, when the positive/desirable response might also refer to performance (i.e., sport) or physical recovery. Responding to the research priorities of the Australian Army we set out to develop an instrument that would assess short term *performance capability* - to capture the desired resilience profile of responding to change-and-adversity that their personnel should demonstrate (Gilmore, 2016). Accordingly, this conceptualisation of resilience should encapsulate performance capability: (a) persisting/thriving through challenging situations; (b) rebounding following setbacks; (c) mitigating detrimental effects if-and-when they occur; and (d) learning and improving from challenges – including deliberate and planned challenges (e.g., training) as well as unexpected or unplanned challenges (ranging from the theater-of-war to also include family issues, interpersonal conflict and equipment failures – cf. Richardson, 2002 - see also Forces Command Resilience Plan, 2015). This focus on the *dynamic* maintenance of individual and group/team capability through various circumstances may represent a different definition of resilience to some offered in the literature, some of which focus on relatively stable attributes of the individual Indeed, The Australian Army currently defines resilience as “the capacity of individuals, teams and organizations to adapt, recover and thrive in situations of risk, challenge, danger, complexity and adversity” (Forces Command Resilience Plan, 2015). Another key difference between “resilient force capability” and previous work on resilience is the emphasis on overall performance capability as opposed to health, mental health, and stress coping – the traditional foci of resilience research. Nevertheless, the elements of a dynamic process (Luthar et al., 2000) responding to different forms of challenge or adversity (Rutter, 1987; Lee and Cranford, 2008) and a repertoire of resources and tendencies (Agaibi and Wilson, 2005) all remain consistent with resilience research.

To better reflect this emphasis on resilient *performance capability*, we focused instead on the concept of “readiness,” to reflect an acute, situational state, and assessing perceptions of what can be achieved or attempted in the immediate future (cf. Grier et al., 2012). In this research, we proposed that changes in such a readiness state in response to recent events may, over time, be used to *infer* individual and group resilience. For example, an individual who maintains high levels of readiness and capability through significant challenges could be viewed as more resilient than someone whose readiness was impaired by the same, or even less significant, adversity (similar logic could apply for inferring physical or emotional resilience from acute response profiles). An instrument assessing readiness in this way would not only reflect a response to recent critiques of resilience measurement (e.g., Windle et al., 2011; Pangallo et al., 2015), but it would better facilitate the comparison and analysis of situational psychological *perceptions* to *objective* indices of physiological stress and resilience indicators such as cortisol, testosterone, and heart-rate variability (e.g., Hellewell and Cernak, 2018).

### Defining and Operationalizing Readiness

Based on this proposition that acute subjective ratings of “readiness” represent a potentially useful tool for supporting performance and training, then the next step is to conceptualize acute readiness. We operationally defined “readiness” as *an acute state of preparation and capability to perform any key task or role, in the immediate future*. We expected this readiness state to fluctuate in response to recent tasks, for example those causing fatigue. This conceptualization differs from some established approaches, detailed below, which reflect a more general, chronic development of skills or capability. To be useful for informing immediate performance management and key decision-making, the instrument we developed needed to focus on the acute assessment of readiness.

As an example of how readiness has previously been monitored, the United States Army defined *unit* readiness as “the ability of a unit to perform as designed” (Dabbieri, 2003; p. 28), assessed in four areas: personnel, equipment-on-hand, equipment serviceability, and training (Army Regulation 220-1, 2003). *Personnel readiness* indicated the extent to which key roles in the unit were occupied by trained and capable individuals – e.g., percentages of fulfilled positions, whether they were available for deployment, and whether they were qualified/trained for their assigned positions. *Equipment-on-hand* referred to the extent that the necessary equipment was available to perform the unit’s role/mission: typically measured as a percentage of available-versus-specified equipment. *Equipment readiness* indicated the extent to which the equipment-on-hand was functional and operational. *Training readiness* indicated how soldiers individually, and the unit collectively, are prepared to execute assigned tasks and missions – exampled indices might include commander’s rating of soldier individual performance of wartime tasks, rating of the unit’s collective performance, and estimated number of training days for soldiers and the unit needed to be ready to perform such tasks (Griffith, 2006). Under this approach to monitoring readiness, numerous bureaucratic and administrative records had to be combined, often taking significant time and resources, to build a representation of a unit’s readiness. These evaluations would be, by necessity, infrequent and after-the-fact: relying on a combination of information from diverse sources. Such a combination of measures is not uncommon, and military contexts are often viewed as strong examples of organization and structure. Nevertheless, approaches like this may limit the ability of readiness monitoring tools to inform key decision-making. A more immediate and more readily integrated approach to monitoring may be available through the frequent use of short and psychometrically sound measures.

Where reviews and research have attempted to conceptualize readiness, their focus has been both broad, and longer term, spanning multiple constructs, including: self-efficacy; commitment; perceived organizational support; physical fitness; sense-of-community; technical competence; family-life; and job satisfaction (Adams et al., 2009; Blackburn, 2014). While these narrative reviews offered a broad overview of readiness, they included more stable traits and attributes, and did not offer clear advice for measurement, noting for example: “Measures that do exist are generally tailored to a specific domain and their generalizability is unclear. The models related to individual readiness are still relatively few and often lack empirical support. These have not been broadly validated” (Adams et al., 2009, p. iii). Hence the necessity of developing a suitable psychometric measure of acute readiness is increasingly clear.

### Existing Psychometric Measures of Readiness

Psychometric research studies have examined different forms of readiness, including: exercise readiness (e.g., freshness/energy and fatigue -Strohacker and Zakrajsek, 2016; Strohacker et al., 2021); readiness to return to sport following injury (skills/fitness and confidence/self-efficacy – Conti et al., 2019); cognitive readiness (e.g., operational and strategic – Grier, 2012; Grier et al., 2012); and – considering a military context - readiness for combat (e.g., discipline and “military climate” - Bester and Stanz, 2007; see also Wen et al., 2014). None of the existing scales span all the different roles fulfilled by military personnel - reaching beyond combat - which necessitated the development of a new specific measure. In reviewing such scales, it is clear that “readiness” can be conceptualized as multidimensional, spanning multiple domains including physical, cognitive, emotional, social, skills/training, and even equipment/resources. In combination, these different components may combine to form an overall indication of acute readiness. Further, important distinctions can be made between acute readiness versus attributes developed over time, such as skills-and-training, as well as a distinction between group-level constructs such as climate and individual states such as fatigue or freshness. We set out to develop a scale to focus on acute, individual, multidimensional readiness – in order to (when implemented) detect short-term changes in individual and group capability.

### Objective Measures and Wearables

Aside from time-consuming and resource-heavy medical examinations, the main alternative to a psychometric approach for monitoring readiness would be the use of wearable devices to monitor physiological signals such as heart rate, heart-rate variability, skin temperature, and galvanic skin response (Domb, 2019; Seshadri et al., 2019). Biochemical markers are also available through the sampling of sweat and sometimes blood to capture metabolites and electrolytes (Bandodkar and Wang, 2014; Lee et al., 2018). Decreasing production costs, increasing portability and the opportunity for real-time monitoring has made these popular options. Nevertheless, these devices can demonstrate inconsistent reliability between different circumstances, and are often dependent on communications network, with implications for battery life and data-security (Evenson et al., 2015; Baig et al., 2017; Peake et al., 2018; Seshadri et al., 2019). In many settings, including the military, construction, food processing, nursing and allied health professions, complications are caused via: (a) exposure to wide variations in temperatures; (b) frequent heavy usage; (c) impacts; and (d) challenges such as water, sweat, grit and sand. Keeping such devices operational over long periods can generate a significant impost, for example, through charging, maintenance and software updates. The information extracted from the user is often removed, analyzed and stored elsewhere, which may not ‘empower’ the user in terms of increasing awareness or facilitating immediate decision making (Seshadri et al., 2019). Without access to suitable network connectivity or local information processing capabilities, the raw information gathered by these devices is often uninterpretable by the user, and over extended periods may simply be deleted or become outdated before it is uploaded. There are also reported occasions where a user’s own perceptions and performance differ substantially from the interpretation offered by wearables, such as athletes being removed from competition despite feeling good and performing well (Blair et al., 2017; Coyne et al., 2018). For these reasons, coupled with the inability of wearables to evaluate all dimensions of readiness (for example skills/training, team-functioning an equipment), a “low-tech” psychometric instrument may be beneficial at least to complement wearable technology: both to compensate in instances when conditions limit the effectiveness of the wearable, and also in facilitating better alignment between subjective perceptions and objectively measured data.

### Delineating Acute Readiness From Related Concepts

Finally, in reviewing existing psychometric measures of relevant and related concepts, several key issues support the development of a new instrument. Measures such as stress-recovery (e.g., (Kölling et al., 2015; Nässi et al., 2017), daily hassles (e.g., (Holm and Holroyd, 1992) and the Task-Load Index (e.g., Hart and Staveland, 1988; Byers et al., 1989) all assess subjective perceptions of recent *past* events. These measures do not assess perceptions of what a person is able to do in the immediate future, and while we may expect a close relationship, performers in many settings must often complete challenging tasks but remain “ready” for another challenge – indeed this is a core requirement of the role. Measures of current subjective state such as affect (e.g., Watson et al., 1988; Crawford and Henry, 2010), anxiety or wellbeing (Ryff, 1989; Van Dierendonck, 2005) likewise do not assess the perception of what may be attempted in the immediate future - what one is ready for. Similarly, while there may be a relationship, many personnel (especially in military) are asked to perform regardless of affect or anxiety, and so a more meaningful question would be: “what are you ready for *right now*?.” The ability to gain insights into this “readiness” state would be invaluable to individuals and their managers, for whom planning of actions and responses is often time-limited (for example, 2-to-4 h was typical in Burr, 2018). The other related concept might be self-efficacy (Bandura, 1977; Sherer et al., 1982): which can be applied to specific tasks or broader skill-sets. While clearly relevant to the concept of readiness, especially with reference to roles and skills, we argue that immediate readiness has a more specific focus than the broad judgment of being capable that typically informs self-efficacy. Likewise, self-efficacy is more likely to be relatively stable over time (Ryckman et al., 1982; Lane et al., 2004; Chesney et al., 2006), indicating broad perceptions of capability, but not reflecting immediate judgments of situational readiness as a function of recent events such as sleep, nutrition, trauma, or physical and mental fatigue. Responding to these concerns, we developed a simple instrument that would be meaningful to users and their immediate line-managers: thus necessitating the use of plain language in both the questions/items and the higher-level constructs.

### Present Research

A systematically developed measure of acute readiness, with items that are applicable across diverse professions and job roles, is necessary for timely, informative and psychometrically sound assessments of readiness. We aimed to develop and evaluate the initial evidence of validity for an instrument we called the Acute Readiness Monitoring Scale (ARMS). The ARMS was designed as a multidimensional measure assessing individuals’ perceptions of readiness for imminent challenges, drawing on capabilities from the domains of physical, cognitive, emotional, social, skills-training and equipment. We collected data from personnel across all three phases of the Army’s “Force Generation Cycle^1^,” spanning diverse roles and ranks, and then conducted two analyses to assess the internal structure (to determine the extent to which the items of a measurement instrument are in line with the construct of interest via factor analyses; Chan, 2014). We also sought to evaluate the correlations of ARMS factors to other related variables. These steps are in accordance with the Standards for Educational and Psychological Testing (The Standards; developed by the American Educational Research Association [AERA] et al., 2014). Additionally, we sought to examine evidence for reliability and discriminant validity of the subscales of the ARMS.

## Overview of Study 1

The aim of Study 1 was to first develop a pool of items to assess acute readiness in the form of: (a) overall readiness; (b) physical readiness; (c) cognitive readiness; (d) emotional readiness (termed “readiness for threat-and-challenge” to be more acceptable to the user-group); (d) the social readiness of the unit, group or team; (e) the suitability of one’s training and skills for immediate tasks; and (f) equipment readiness. Second, we set out to evaluate the internal structure, internal consistency, and discriminant validity of the subscale scores of the new measure.

### Method – Study 1

#### Participants

We targeted a sample of five to ten participants per item (Anthoine et al., 2014), acknowledging that (Comrey and Lee, 1992) asserted that a sample size 300 is typically viewed as appropriate. The total final sample consisted of 770 Australian Army personnel (*N*_male_ = 677, *N*_female_ = 93), with a mean age of 26.5 years (SD = 7.0 years). Seven participants declined to participate at the informed consent stage, and any corresponding data were destroyed. One participant spoiled their answers and was excluded. Participants were drawn from all three phases of the “Force Generation Cycle” (*N*_ready_ = 358; *N*_readying_ = 186; *N*_reset_ = 226) and from a wide variety of trades, spanning: infantry, artillery, engineers, signals, chefs, armored divisions, clerks, and more, but not including special forces. Participants were drawn from a wide range of ranks (*N*_PR_ = 572; *N*_PR(P)_ = 15; *N*_LCPL_ = 46; *N*_CPL_ = 71; *N*_SGT_ = 18; *N*_WO__2_ = 16; *N*_WO__1_ = 1; *N*_LT_ = 10; *N*_CAPT_ = 6; *N*_MAJ_ = 13; *N*_LTCOL_ = 1). Career length in the Army ranged from 0.5 – 42.5 years (mean = 5.2 years, SD = 5.5 years). Ethnicity was self-reported using the Australian Bureau of Statistics reporting system (Australian Bureau of Statistics, 2016), coded as follows: (a) Aboriginal and Torres Strait Islander (*n* = 40); (b) Arab, North Africa and Middle East (*n* = 11); (c) Africa (*n* = 5); (d) Americas – North and South (*n* = 4); (e) Asia (*n* = 31); (f) Caucasian and Western European (*n* = 570); (g) Melanesia, Polynesia and New Zealand Peoples (*n* = 12); (h) South and Eastern European (*n* = 14); (i) Mixed/Other (*n* = 24); and (j) no answer (59) Table 1. The total sample was randomly divided into *n* = 500 for the Exploratory Factor Analysis (Study 1), and *n* = 270 for the Confirmatory Factor Analysis (Study 2) and this split was checked to ensure no significant differences in composition. The subsequent evaluation of convergent and divergent validity was conducted using the whole sample.

**TABLE 1:** Summary of participant characteristics.

Participant characteristic	Classification	Frequency (n)	Mean (SD)
Sex	Female	93	
	Male	677	
Age	Years		26.5 (6.99)
Career Length	Years		5.22 (5.54)
Force Generation Cycle Phase	Ready – e.g., Trained, equipped and available	320	
	Readying – e.g., Training, equipping, rehearsing	311	
	Reset – e.g., Recovery, debrief, reflect, re-plan	139	
Ethnicity	Aboriginal and Torres Strait Islander	40	
	Caucasian	570	
	South and Eastern Europe	14	
	North Africa, Middle East, Arab Peninsula	11	
	Africa - West, East, Central, South	5	
	Asia	31	
	Melanesia, Polynesia, NZ Peoples	12	
	America - North and South	4	
	Other/Mixed	24	
	Not provided	59	
Highest Level of Education	Some High School	6	
	Year 10 Certificate	75	
	Year 12	406	
	Certificate or Diploma (non-university, e.g., TAFE)	231	
	Tertiary undergraduate/graduate (e.g., BA, BSc)	35	
	Tertiary post-graduate (e.g., MA, MSc, PhD)	8	
	Not provided	9	
Rank	Private	572	
	Private (proficient)	15	
	Lance Corporal	46	
	Corporal	71	
	Sergeant	18	
	Warrant Officer 2	16	
	Warrant Officer 1	1	
	Lieutenant	10	
	Captain	6	
	Major	13	
	Lt Colonel	1	

##### Acute Readiness in Monitoring Scale

The ARMS items were designed to assess participants’ perceptions of readiness for immediate challenges and tasks that could be demanding physically, cognitively, emotionally, drawing on specific skills/training, teamwork and team functioning, or equipment. Items were also developed to provide an overall appraisal of readiness. An initial pool of items was developed based upon the operational definition of acute readiness. These items were then reviewed by the rest of the research team, who made suggestions for improvements and/or proposed alternative items. Items were kept brief (but not single word items), were not double-barreled in syntax, and did not borrow heavily from any one existing measure. Reverse-scored items were included. The content of items was informed by existing self-report measures of readiness, affect, perceived task load, stress-recovery, coping, and fatigue (e.g., stress recovery – Kölling et al., 2015; Task-Load Index – Byers et al., 1989; affect – Watson et al., 1988; wellbeing – Ryff, 1989; self-efficacy – (Sherer et al., 1982). The initial item pool is listed in Supplementary File 1. The items were scored on a seven-point Likert scale from zero (does not apply at all) to six (fully applies), and introduced with the phrase “Please answer the following questions in relation to how ready you feel for any upcoming task or challenge.” The 7-point response format is common in sport and performance psychology (e.g., Bartholomew et al., 2011; Ng et al., 2011) and consistent with survey takers’ preferences: performing well in terms of their discriminative power (Preston and Colman, 2000). Through this process, our team generated 12–14 items assessing perceptions of each construct, with the intention of selecting approximately four items per factor/subscale in the final product. The proposed items were subsequently evaluated by a user group (*n* = 23) and an international panel of experts (*n* = 7) – with feedback leading us to (for example) de-emphasize resilience concepts (initially a focus from the industry stakeholders), and remove references to “right now” which was already implied in the question stem. We also presented and discussed the proposed approach to a group of senior stakeholders at a workshop held on 8th April 2019.

### Procedure

Ethical approval was obtained for both the expert panel consultation (HREC, 1739) and the main data collection (DST LD 08-19 and HREC 2193). Subsequently, task orders were issued from Australian Army Headquarters to make troops available for data collection visits lasting approximately 1-h. Data-collection took place in person, at locations across Australia, using paper-and-pen surveys – typically in groups of between 15 and 100 personnel in one sitting. Commanding officers were not required to be present, but some chose to attend and complete the task with their units (see Participants, above). Prior to survey administration, participants were advised of the wider intention of the project to generate a readiness monitoring instrument and shown mock-ups of how such a system could look and be used in practice. Participants were assured that were no right or wrong responses, reminded of the anonymity of their responses, and encouraged to respond honestly. We recorded the time of day that survey completion began, as well as the most recent task that the participant had completed, as well as how demanding they found that using the NASATask Load Index (TLX). Only personnel who were on-base at the time of data collection were included. Participation in the study was voluntary, and this was emphasized through the informed consent process as well as in the small presentation preceding the data collection. All participants completed a written informed consent form prior to taking the survey, which was administered in person immediately prior to the data collection. Participants were informed they could return to other tasks if they did not wish to participate, although – as implied above - several chose to complete the survey with their team and then withheld consent for the data to be used.

#### Data Analyses

Exploratory factor analysis (EFA) identifies the dimensionality of constructs by examining relations between items and factors (Netemeyer et al., 2012). For this reason, EFA is typically performed in the early stages of developing a new or revised instrument (Wetzel, 2012). In this study, seven candidate factors were developed: (a) overall readiness (b) physical readiness; (c) cognitive readiness (d) threat-challenge readiness; (e) skills-readiness; (f) group readiness; and (g) equipment readiness. This hypothesis was used to inform how we explored the structural pattern of the preliminary scale, along with a scree plot and eigenvalues (Thompson, 2004). Scree plots are useful to estimate where a significant drop occurs in the strength of possible factors (Cattell, 1966; Netemeyer et al., 2003).

We developed the factorial structure of the new measure using EFA, Exploratory Structural-Equation Modeling (ESEM) and CFA. ESEM incorporates aspects of the CFA process, such as specifying item-combinations and relationships, within the exploratory phase of the scale development (Asparouhov and Muthén, 2009). As such, ESEM is useful in clarifying key issues such as cross loading and potential shared error variance before moving to the CFA stage. Statistical analyses were conducted in SPSS (IBM Corp, 2016 – data cleansing, collating emerging factors, checking, internal reliability) and MPlus 8.3 (Muthén and Muthén, 2012 - running models, assessing each factor, model, and fit indices). We worked from the Pearson correlation matrix that is the default in MPlus, and we used oblique “geomin” rotation in line with guidance for EFA (Dien, 2010). For CFA modeling, latent factors were permitted to correlate, with cross-loadings of items on unintended factors being constrained to zero. Similar to CFA, as the analysis progressed into evaluating ESEM models, items could load on their predefined latent factors, while estimating cross loadings and considering this in the development of the evolving model.

As complete models were evaluated, goodness-of-fit was evaluated using the χ^2^ test (with *p*-value) and χ^2^/df (degrees of freedom), Comparative Fit Index (CFI), Tucker-Lewis index (TLI), Root Mean Square Error of Approximation (RMSEA), and Standardized Root Mean Square (SRMR). Adequate and excellent model-to- data fit was indicated by non-significant χ^2^ test or χ^2^/df < 2.5, CFI and TLI values >0.90 and 0.95, respectively, and RMSEA and SRMR values of or <0.08 and 0.06, respectively (Hu and Bentler, 1999; Marsh et al., 2004; Hooper et al., 2008). The strength of factor loadings was informed by the recommendations put forth by Comrey and Lee (1992) – i.e., > 0.71 = “excellent,” > 0.63 = “very good,” > 0.55 = “good,” > 0.45 = “fair,” < 0.30 = “poor”). The internal consistency of the subscale scores was determined through an assessment of Cronbach’s alpha (Cronbach, 1951). In line with the recommendation by Nunnally (1978), internal consistency estimates > 0.70 were deemed adequate.

### Results – Study 1

#### Item Distribution

Prior to the factor analyses, data were scanned for univariate normality regarding the assumption for the use of maximum likelihood estimation method. Median values for skewness and kurtosis for the 88 candidate items were 0.69 and 0.11, respectively, and ranged from −2.21 to 1.36 for skewness, and −1.01 to 5.16 for kurtosis. Two heavily skewed items were removed from the analysis (“I am sick today” and “I am injured today” – both very frequently scored as zero or “zero inflated”). We observed up to 2% missing data in some variables, and so data were analyzed using a robust maximum likelihood estimator (MLR). MLR yields robust fit indices and standard errors in the case of non-normal data and operates well when categorical variables with a minimum of five response categories are employed (Rhemtulla et al., 2012; Bandalos, 2014).

#### Configurations of the Proposed Factors

The exploratory factor analysis, using the subset of *n* = 500 respondents, process began with an initial analysis run to obtain eigenvalues for each factor in the data. Next, the Kaiser-Meyer-Olkin (KMO) Measure of Sampling Adequacy (KMO = 0.962) test and Bartlett’s Test of Sphericity (χ^2^(3655) = 37578.3, *p* < 0.001) were employed to determine that the data collected were appropriate for an exploratory factor analysis. Having established the data were suitable for EFA, we continued to explore the data. The initial analysis suggested 13 factors with an eigenvalue over one, whereas the scree plot suggested a firm transition (“scree”) after nine factors. As such, we used MPlus to generate factor solutions between 7 and 14 factors, and reviewed the solutions in relation to: (a) the hypothesized factor structure; (b) feedback from the expert panel (prioritizing conceptual clarity); and (c) user-group workshop and stakeholder inputs (for example prioritizing interpretability, brevity and parsimony). By reviewing the multiple factor solutions simultaneously, we recorded which items consistently loaded together, and which items consistently cross-loaded, or failed to load meaningfully. Items largely loaded consistently into the expected factors, but two proposed factors in particular – physical readiness and cognitive readiness – contained two clusters of items loading meaningfully onto separate factors: that we interpreted to distinguish between “readiness” and “fatigue.” We investigated this development further using the process detailed below. The proposed threat-challenge factor split into items clearly pertaining to “readiness” alongside others capturing simply “affect,” leading to these latter items to be discarded as they were considered inconsistent with the targeted emphasis on performance capability.

At this stage, we examined each of the hypothesized factors using EFA, ESEM and CFA to systematically remove problematic items, and then re-run the resulting ESEM model with the best performing items. For these analyses, we included CFA steps as they were helpful in comparing models and selecting items with strong primary factor loadings to ultimately inform the final ESEM model (see also, Bhavsar et al., 2020). Model misspecification was identified through assessments of standardized factor loadings and modification indices, in a manner similar to item reduction approaches used in previous scale development procedures (e.g., Rocchi et al., 2017). Alongside these statistical criteria, we also considered the conceptual coverage of the items. Items with standardized factor loadings below 0.30, as well as items with multiple (two or more) moderate-sized or large modification indices (over 10) were reviewed and considered for deletion. As such, 56 of the 88 items were deleted in a systematic manner over several iterations. The resulting nine one-factor models each had excellent fit (see Table 2). After removing one item that cross loaded (SK3), the revised combined/overall model demonstrated good fit [χ^2^ (428) = 1034.867, *p* < 0.001; χ^2^/428 = 2.4; CFI = 0.95; TLI = 0.95; SRMR = 0.05; RMSEA = 0.05 (90% CI: 0.05, 0.06)]. The result of EFA in Study 1 was a refined measure that included 32 items arranged into nine factors (see Table 3).

**TABLE 2:** Model fit indices of different models developed during the ESEM process.

Model	*X2*	*P*	df	CFI	TLI	SRMR	RMSEA	[90% CI]
Full Readiness EFA – 7 factors (hypothesized) – all items (First run)	10245.535	<0.001	3233	0.822	0.789	0.32	0.066	0.064 – 0.067
Full Readiness EFA – 10 factors (PH, CO and TC split) – all items	7751.601	<0.001	2993	0.879	0.845	0.025	0.056	0.055 – 0.058
Overall Readiness – One factor – all items	908.962	<0.001	54	0.814	0.773	0.65	0.178	0.168 – 0.188
Overall Readiness – One factor with 6,7,8,12	15.213	0.0005	2	0.989	0.968	0.016	0.115	0.066 – 0.172
**Overall Readiness – One factor with 3,6,7,8**	**2.749**	**0.253**	**2**	**0.999**	**0.998**	**0.010**	**0.027**	**0.000 – 0.097**
Physical Readiness – One factor – all items	1274.534	<0.001	54	0.679	0.608	0.143	0.213	0.203 – 0.223
Physical Readiness – Two factor – all items	244.834	<0.001	43	0.947	0.919	0.036	0.097	0.085 – 0.109
Physical Readiness – Two factors, best performing items (+2,4,7 and -5,6,9,10)	7.090	0.5270	8	1.000	1.001	0.008	0.000	0.000 – 0.048
**Physical Readiness – Two factors, best performing items (+2,3,4 and -5,6,9,10)**	**13.852**	**0.0857**	**8**	**0.997**	**0.993**	**0.011**	**0.038**	**0.000 – 0.071**
Cognitive Readiness – One factor – all items)	865.279	<0.001	54	0.825	0.787	0.081	0.173	0.163 – 0.184
Cognitive Readiness – Two factor – all items)	221.154	<0.001	43	0.962	0.941	0.027	0.091	0.079 – 0.103
**Cognitive Readiness – Two factors, best performing items (+2,3,4 and -6,7,8)**	**12.124**	**0.0164**	**4**	**0.996**	**0.986**	**0.007**	**0.064**	**0.025 – 0.106**
Threat-Challenge Readiness – One factor – all items	842.326	<0.001	77	0.804	0.769	0.70	0.141	0.132 – 0.150
Threat-Challenge Readiness – Two factor – all items 1,2,3,4,6,8,9 = affect/state → discard 5,7,10,11,12,13,14 = capability	457.968	<0.001	64	0.899	0.857	0.41	0.111	0.102 – 0.121
Threat-Challenge Readiness – One factor – Capability	70.937	<0.001	14	0.967	0.951	0.033	0.090	0.070 – 0.112
**Threat-Challenge Readiness – One factor – Best performing Items (11,12,13,14)**	**3.448**	**0.1784**	**2**	**0.999**	**0.996**	**0.011**	**0.038**	**0.000 – 0.104**
Group-Team Readiness – One factor – all items	590.837	<0.001	54	0.909	0.889	0.042	0.141	0.131 – 0.152
**Group-Team Readiness – One factor – Best performing Items (4,7,8,10)**	**5.460**	**0.0652**	**2**	**0.998**	**0.994**	**0.006**	**0.059**	**0.000 – 0.121**
Skills-Training Readiness – One factor – all items	687.495	<0.001	54	0.853	0.821	0.058	0.153	0.143 – 0.164
**Skills-Training Readiness – One factor – Best Performing Items – 3^††^,5,8,12)**	**0.146**	**0.9294**	**2**	**1.000**	**1.006**	**0.003**	**0.000**	**0.000 – 0.027**
Equipment Readiness – One factor – all items	1826.231	<0.001	77	0.646	0.582	0.097	0.214	0.205 – 0.222
**Equipment Readiness – One factor – best performing items (1,2,3,4)**	**0.353**	**0.8382**	**2**	**1.000**	**1.005**	**0.003**	**0.000**	**0.000 – 0.051**
**Combined overall model EFA (9-factors)**	**444.781**	**<0.001**	**244**	**0.984**	**0.968**	**0.012**	**0.041**	**0.035 – 0.047**
**Combined overall model ESEM (9-factors)**	**1034.867**	**<0.001**	**428**	**0.952**	**0.945**	**0.045**	**0.053**	**0.049 – 0.057**
***Combined overall model CFA (NEW DATASET 9-factors)	**902.363**	**<0.001**	**428**	**0.936**	**0.925**	**0.057**	**0.064**	**0.058 – 0.070**

*χ2 = Chi-square test of exact fit; CFI = Comparative Fit Index; TLI = Tucker–Lewis index; RMSEA = Root Mean Square Error of Approximation; 90% CI = 90% confidence interval of the RMSEA; ESEM = exploratory structural equation modeling. ^††^ = dropped due to cross loading when incorporated into full 9-factor model.*

**TABLE 3:** Factor loadings for the ARMSS from study 1.

	Original item code	Item wording	Overall readiness	Physical readiness	Physical fatigue	Cognitive readiness	Cognituve fatigue	Thread-Challenge readiness	Group-Team readiness	Skills-Training readiness	Equipment readiness
1	OV3	I feel ready to deal with serious threats	**0.620**	0.198	−0.008	0.031	0.127	0.037	−0.002	0.009	0.003
2	OV6	My skills and experience make me capable of meeting any challenge	**0.711**	0.016	0.054	−0.037	−0.021	−0.022	0.053	0.084	0.080
3	OV7	I feel ready to deal with uncertainty	**0.789**	−0.005	0.042	0.112	0.014	0.026	0.036	−0.029	0.042
4	OV8	I feel confident in taking control of situations	**0.680**	0.005	−0.065	0.027	−0.006	0.149	−0.036	0.124	−0.052
5	PH2	I am physically fit	−0.025	**0.858**	0.021	0.018	−0.001	0.051	−0.007	0.013	0.012
6	PH3	I am physically prepared	0.014	**0.959**	−0.011	−0.024	0.033	0.025	0.005	0.065	−0.011
7	PH4	I am physically fresh	0.054	**0.493**	0.222	0.303	−0.047	−0.038	0.038	−0.065	0.02
8	PH5R	I am physically tired	−0.004	0.032	**0.760**	0.077	−0.012	0.034	−0.019	−0.048	0.057
9	PH6R	My muscles are sore	0.052	−0.093	**0.689**	−0.117	0.025	0.069	0.032	0.009	−0.021
10	PH9R	I am fatigued	−0.065	0.014	**0.903**	0.008	0.02	0.015	0.007	0.033	0.014
11	PH10R	I am physically spent	0.041	0.042	**0.656**	0.003	0.260	−0.136	−0.016	0.098	−0.059
12	CO2	I can focus well	0.009	−0.02	0.014	**0.695**	0.015	0.209	0.049	0.011	0.019
13	CO3	I am mentally prepared	0.122	0.064	−0.011	**0.758**	0.037	0.021	0.002	0.065	0.001
14	CO4	I am thinking clearly	0	−0.006	0.026	**0.577**	0.110	0.252	−0.027	0.064	−0.005
15	CO6R	I am mentally tired	−0.065	0.006	0.295	0.084	**0.533**	0.044	0.019	−0.056	0.069
16	CO7R	My mind is fuzzy today	0.052	0.009	0.02	−0.014	**0.927**	−0.018	−0.01	0.008	0.032
17	CO8R	I cannot focus today	0.003	−0.028	0.008	0.036	**0.748**	0.124	0.032	0.022	−0.048
18	TC11	I am ready to process significant problems	0.061	−0.007	0.102	0.005	0.009	**0.700**	0.022	0.048	−0.021
19	TC12	No matter the challenge, I am ready for it	0.165	0.163	0	0.01	−0.023	**0.656**	0.021	−0.015	0.011
20	TC13	I have things under control today	−0.039	0.042	−0.002	0.078	0.033	**0.711**	0.008	0.088	0.019
21	TC14	I can handle unpleasant feelings	0.063	0.027	0.007	0.016	0.05	**0.679**	0.007	−0.021	0.059
22	GP4	My team is ready	0.134	−0.01	−0.007	0.01	0.046	−0.045	**0.800**	−0.027	0.081
23	GP7	My team has strong systems and processes	−0.04	−0.014	0.063	0.052	−0.064	0.041	**0.842**	0.007	0.013
24	GP8	My team works well together	0.027	0.033	−0.003	−0.011	0.018	−0.001	**0.908**	0.002	−0.017
25	GP10	I have confidence in my team	−0.014	0.017	−0.022	−0.024	0.006	0.033	**0.908**	0.056	−0.031
26	SK5	I offer significant value to my role/Unit	0.057	0.073	0.011	0.041	−0.006	0.053	−0.013	**0.642**	0.046
27	SK8	I am capable of delivering my role	−0.029	0.024	−0.005	−0.015	0.084	−0.019	0.029	**0.774**	0.023
28	SK12	I feel confident in my abilities to perform my role	0.128	−0.035	0.033	0.064	−0.033	0.037	0.035	**0.738**	0.004
29	EQ1	I have all the equipment I need	−0.026	0.037	0.002	0.081	−0.002	0.001	0.018	−0.01	**0.792**
30	EQ2	My equipment is well-maintained	−0.012	0.014	−0.063	0.045	0.004	−0.007	0.115	0.083	**0.727**
31	EQ3	My equipment is fit-for-purpose	0.031	−0.003	0.015	−0.095	0.057	0.083	−0.009	−0.007	**0.891**
32	EQ4	My equipment is world-leading	0.051	0.025	0.041	0.042	0.002	0.066	0.03	0.02	**0.714**

*Significant factor loadings are represented by 8, and bold font for main factor. Negligible loadings are pale gray, with those >0.2 being dark gray.*

Standardized factor loadings were significant and above 0.30 (range 0.49 to 0.90; see Table 3). Four cross-loadings greater than 0.20 on unintended factors were present, and this was not viewed as a concern warranting their deletion (see also Bhavsar et al., 2020). Subscale correlations ranged from 0.26 to 0.77 and were in the expected directions (see Tables 4, 5). Cronbach’s alpha reliability coefficients are also reported in Tables 4, 5. These were over 0.70 for all factors.

**TABLE 4:** Correlation table for concurrent and discriminant validity of study 2 – acute and short-term correlates.

	Variable	1	2	3	4	5	6	7	8	9	10	11	12	13	14	15	16	17	18	19	20
1	Overall Readiness	–																			
2	Physical Readiness	0.55** (0.47–0.63)	–																		
3	Physical Fatigue (R)	0.32** (0.26–0.37)	0.44** (0.36–0.52)	–																	
4	Cognitive Readiness	0.67** (0.63–0.71)	0.56** (0.47–0.63)	0.46** (0.37–0.54)	–																
5	Cognitive Fatigue (R)	0.40** (0.34–0.45)	0.35** (0.25–0.44)	0.61** (0.54–0.67)	0.65** (0.58–0.72)	–															
6	Threat-Challenge Readiness	0.68** (0.64–0.72)	0.59** (0.50–0.66)	0.43** (0.35–0.50)	0.77** (0.71–0.80)	0.57** (0.50–0.64)	–														
7	Group-Team Readiness	0.41** (0.35–0.46)	0.36** (0.27–0.44)	0.31** (0.22–0.39)	0.44** (0.34–0.52)	0.34** (0.24–0.42)	0.43** (0.35–0.51)	–													
8	Skills-Training Readiness	0.68** (0.63–0.71)	0.44** (0.35–0.52)	0.28** (0.19–0.37)	0.60** (0.53–0.68)	0.35** (0.26–0.44)	0.59** (0.51–0.67)	0.46** (0.46–0.55)	–												
9	Equipment Readiness	0.40** (0.35–0.45)	0.34** (0.26–0.42)	0.26** (0.16–0.36)	0.38** (0.30–0.46)	0.24** (0.15–0.32)	0.34** (0.25–0.42)	0.41** (0.32–0.49)	0.33** (0.23–0.42)	–											
10	Supervisor Rating	0.15** (0.07–0.22)	0.14^††^ (0.01–0.26)	0.04 (0.07–0.14)	0.09 (−0.03–0.20)	0.02 (−0.09–0.12)	0.12 (0.01–0.25)	0.01 (−0.11–0.12)	0.17** (0.06–0.28)	0.03 (−0.07–0.13)	–										
11	TLX Mental Demand	0.01 (−0.06–0.09)	−0.04 (−0.13–0.06)	−0.09^††^ (−0.17–0.00)	−0.03 (−13–0.07)	−0.06 (−0.15–0.04)	0.01 (−0.10–0.10)	0.00 (−0.12–0.13)	0.01 (−0.12–0.14)	0.01 (−0.09–0.09)	0.09 (−0.03–0.18)	–									
12	TLX Physical Demand	0.05 (−0.02–0.10)	0.11^††^ (0.02–0.20)	−0.04 (−0.13–0.06)	0.03 (−0.06–0.12)	−0.04 (−0.13–0.06)	0.07^††^ (−0.02–0.16)	0.06 (−0.04–0.15)	0.06 (−0.04–0.15)	0.00 (−0.10–0.09)	−0.08 (−0.16–0.03)	0.33** (0.16–0.46)	–								
13	TLX Time Pressure	−0.01 (−0.11–0.09)	−0.06 (−0.16–0.03)	−0.16** (−0.25 to −0.06)	−0.09** (−0.19–0.01)	−0.13** (−0.22 to −0.03)	−0.02 (−0.11–0.07)	−0.04 (−0.14–0.06)	0.03 (−0.06–0.14)	−0.04 (−0.15–0.05)	0.06 (−0.05–0.16)	0.50** (0.31–0.64)	0.41** (0.33–0.50)	–							
14	TLX Effort	0.09 (−0.01–0.17)	0.03 (−0.08–0.12)	−0.07^††^ (−0.05–0.03)	0.01 (−0.08–0.10)	−0.04 (−0.12–0.06)	0.08^††^ (−0.02–0.17)	0.11^††^ (0.00–0.20)	0.11^††^ (0.00–0.20)	0.06 (−0.04–0.16)	−0.05 (−0.15–0.06)	0.46** (0.29–0.60)	0.49** (0.41–0.57)	0.55** (0.47–0.62)	–						
15	TLX Frustration	−0.13** (−**0.19 to**−**0.07)**	−0.19** (−**0.28 to**−**0.1)**	−0.29** (−**0.37 to**−**0.20)**	−0.21** (−**0.30 to**−**0.13)**	−0.22** (−**0.3- to**−**0.13)**	−0.23** (−**0.31 to**−**0.14)**	−0.18** (−**0.28 to**−**0.08)**	−0.14** (−**0.23 to**−**0.04)**	−0.17** (−**0.26 to**−**0.07)**	0.00 (−0.10–0.11)	0.41** (0.32–0.51)	0.20** (0.10–0.29)	0.53** (0.44–0.60)	0.35** (0.26–0.45)	–					
16	Positive Affect	0.42** (0.33–0.50)	0.46** (0.37–0.53)	0.32** (0.22–0.40)	0.47** (0.39–0.54)	0.36** (0.28–0.44)	0.52** (0.45–0.59)	0.33** (0.23–0.42)	0.39** (0.29–0.46)	0.32** (0.23–0.40)	−0.01 (−0.12–0.09)	0.04 (−0.07–0.15)	0.13^††^ (0.03–0.22)	0.01 (−0.08–0.11)	0.14^††^ (0.04–0.23)	0.15^††^ (−0.24 to −0.04)	–				
17	Negative Affect	−0.27** −**(0.37 to**−**0.16)**	−0.21** −**0.31 to**−**0.10)**	−0.33** (−**0.41 to**−**0.24)**	−0.40** (−**0.48 to**−**0.30)**	−0.44** (−**0.52 to**−**0.35)**	−0.38** (−**0.47 to**−**0.31)**	−0.24** (−**0.34 to**−**0.12)**	−0.27** (−**0.36 to**−**0.17)**	−0.18** (−**0.27 to**−**0.08)**	−0.15** (−**0.27 to**−**0.04)**	0.14^††^ (0.03–0.23)	0.08^††^ (−0.03–0.17)	0.15** (0.05–0.25)	0.07^††^ (−0.02–0.15)	0.26** (0.16–0.34)	−0.08^††^ (−0.17–0.02)	–			
18	K10 Distress	−0.38** (−**0.47 to**−**0.28)**	0.36** (−**0.44 to**−**0.27)**	−0.40** (−**0.47 to**−**0.32)**	−0.50** (−**0.58 to**−**0.42)**	−0.52** (−**0.59 to**−**0.44)**	−0.49** (−**0.57 to**−**0.42)**	−0.31** (−**0.40 to**−**0.19)**	−0.39** (−**0.49 to**−**0.29)**	−0.24** (−**0.34 to**−**0.16)**	−0.16** (−**0.29 to**−**0.05)**	0.11^††^ (−0.02–0.21)	0.06 (−0.03–0.16)	0.13^††^ (0.03–0.22)	0.04 (−0.06–0.13)	0.29** (0.20–0.38)	−0.31** (−**0.40 to**−**0.22)**	0.67** (0.59–0.75)	–		
19	Time of Day	0.03 (−0.06 −0.11)	−0.01 (−0.10–0.09)	0.01 −0.08–0.10	−0.04 (−0.14–0.05)	−0.09 ^††^ (−0.18 to −0.01)	−0.07 (−0.17–0.02)	0.00 (−0.10–0.09)	−0.01 (−0.11–0.08)	0.13** (0.03–0.21)	0.02 (−0.07–0.12)	0.03 (−0.08–0.12)	−0.06 (−0.14–0.04)	0.02 (−0.12–0.07)	−0.01 (−0.1–0.07)	0.04 (−0.05–0.14)	−0.03 (−0.13–0.07)	0.05 (−0.05–0.16)	0.01 (−0.08–0.10)	–	
20	Force Generation Cycle Phase	0.07 (−0.03–0.17)	0.3 (−0.06−0.13)	−0.01 −0.1–0.10	0.05 (−0.04–0.16)	−0.02 (−0.12–0.08)	0.05 (−0.05–0.15)	0.04 (−0.06–0.14)	0.05 (−0.04–0.15)	0.10** (0.01–0.19)	0.18** (0.07–0.28)	0.01 (−0.09–0.08)	−0.12^††^ (−0.22 to −0.03)	−0.07^††^ (−0.16 to 0.02)	−0.04 (−0.13 to 0.05)	−0.04 (−0.13 to 0.05)	−0.03 (−0.13 to 0.07)	−0.10^††^ (−0.20 to 0.00)	−0.10^††^ (−0.21 to 0.00)	0.06 (−0.05 to 0.16)	–
	**Sample**	766	766	765	768	769	770	767	769	764	608	752	752	750	751	751	755	754	746	770	770
	**Cronbach’s Alpha**	0.892	0.830	0.882	0.924	0.869	0.885	0.939	0.856	0.879	n/a	n/a	n/a	n/a	n/a	n/a	0.931	0.898	0.918	n/a	n/a
	**Mean**	17.48	12.40	13.58	13.28	13.06	17.38	15.99	13.63	14.15	13.04	9.71	8.32	10.46	12.41	9.58	30.82	14.99	16.99	1031.87	2.17
	**AVE**	0.493	0.633	0.575	0.464	0.568	0.472	0.749	0.518	0.615	n/a	n/a	n/a	n/a	n/a	n/a	0.619	0.546	0.591	n/a	
	**Skewness/Kurtosis**	−0.53/0.08	−0.63/0.66	−0.05/−0.67	−0.61/0.17	−0.59/−0.46	−0.56/0.06	−0.71/0.24	−0.84/0.85	−0.24/−0.55	−0.58/−0.14	2.59/31.59	0.35/−1.11	−0.92/−0.45	−0.45/−0.51	0.11/−1.25	−0.16/−0.62	1.99/4.90	1.63/2.91	0.72/−1.25	−0.34/−1.55

Cells include r-value with 95% confidence-interval values in parentheses. *Denotes p < 0.05 and **Denotes p < 0.01. We used ^††^ to denote correlations that were statistically significant but with confidence intervals close to including zero. Gray font denotes no meaningful correlation. Black and bold indicates a significant correlation.

**TABLE 5:** Correlation table for concurrent and discriminant validity of study 2 – traits and long-term correlates.

	Variable	1	2	3	4	5	6	7	8	9	10	11	12	13	14	15	16
1	Overall Readiness	–															
2	Physical Readiness	0.55** (0.47–0.63)	–														
3	Physical Fatigue (R)	0.32** (0.26–0.37)	0.44** (0.36–0.52)	–													
4	Cognitive Readiness	0.67** (0.63–0.71)	0.56** (0.47–0.63)	0.46** (0.37–0.54)	–												
5	Cognitive Fatigue (R)	0.40** (0.34–0.45)	0.35** (0.25–0.44)	0.61** (0.54–0.67)	0.65** (0.58–0.72)	–											
6	Threat-Challenge Readiness	0.68** (0.64–0.72)	0.59** (0.50–0.66)	0.43** (0.35–0.50)	0.77** (0.71–0.80)	0.57** (0.50–0.64)	–										
7	Group-Team Readiness	0.41** (0.35–0.46)	0.36** (0.27–0.44)	0.31** (0.22–0.39)	0.44** (0.34–0.52)	0.34** (0.24–0.42)	0.43** (0.35–0.51)	–									
8	Skills-Training Readiness	0.68** (0.63–0.71)	0.44** (0.35–0.52)	0.28** (0.19–0.37)	0.60** (0.53–0.68)	0.35** (0.26–0.44)	0.59** (0.51–0.67)	0.46** (0.46–0.55)	–								
9	Equipment Readiness	0.40** (0.35–0.45)	0.34** (0.26–0.42)	0.26** (0.16–0.36)	0.38** (0.30–0.46)	0.24** (0.15–0.32)	0.34** (0.25–0.42)	0.41** (0.32–0.49)	0.33** (0.23–0.42)	–							
10	CD-RISC – original	0.59** (0.52–0.66)	0.47** (0.38–0.54)	0.27** (0.17–0.36)	0.62** (0.56–0.69)	0.41** (0.31–0.49)	0.64** (0.54–0.71)	0.36** (0.25–0.45)	0.57** (0.48–0.65)	0.36** (0.27–0.43)	–						
11	Stress-mindset	0.26** (0.17–0.35)	0.31** (0.22–0.40)	0.21** (0.12–0.30)	0.32** (0.22–0.41)	0.27** (0.17–0.35)	0.34** (0.25–0.43)	0.21** (0.12–0.31)	0.25** (0.16–0.35)	0.17** (0.07–0.27)	0.43** (0.34–0.51)	–					
12	Self-control	0.34** (0.25–0.43)	0.30** (0.21–0.48)	0.32** (0.24–0.40)	0.37** (0.27–0.44)	0.36** (0.28–0.43)	0.40** (0.32–0.48)	0.24** (0.14–0.32)	0.33** (0.22–0.42)	0.25** (0.17–0.33)	0.47** (0.39–0.54)	0.30** (0.20–0.38)	–				
13	DISC-Q ΣCognitive	−0.03 (−0.12–0.06)	−0.13^††^ (−0.23 to −0.05)	−0.13^††^ (−0.22 to −0.03)	−0.15^††^ (−0.24 to −0.07)	−0.16^††^ (−0.25 to −0.07)	−0.10^††^ (−0.19 to −0.02)	−0.16^††^ (−0.25 to −0.06)	−0.05 (−0.15 to −0.05)	−0.20** (−0.29 to −0.12)	−0.06 (−0.15–0.04)	−0.12^††^ (−0.22 to −0.02)	−0.04 (−0.14–0.05)	–			
14	DISC-Q ΣEmotion	−0.14** (−0.22 to −0.04)	−0.21** (−0.30 to −0.12)	−0.25** (−0.35 to −0.17)	−0.30** (−0.37 to −0.21)	−0.28** (−0.36 to −0.20)	−0.23** (−0.31 to −0.14)	−0.35** (−0.43 to −0.26)	−0.18** (−0.27 to −0.09)	−0.29** (−0.38 to −0.21)	−0.20** (−0.29 to −0.11)	0.20** (−**0.30 to**−**0.10)**	−0.18** (−**0.27 to**−**0.09)**	0.58** (0.52–0.65)	–		
15	Σ Daily Hassles	−0.23** (−0.33 to −0.11)	−0.28** (−0.39 to −0.16)	−0.35** (−0.42 to −0.26)	−0.38** (−0.47 to −0.28)	−0.39** (−0.48 to −0.31)	−0.34** (−0.42 to −0.25)	0.30** (−0.39 to −0.20)	0.26** (−0.37 to −0.15)	−0.21** (−0.30 to −0.13)	−0.31** (−0.43 to −0.21)	−0.29** (−**0.38 to**−**0.22)**	−0.23** (−**0.31 to**−**0.16)**	0.23** (0.15–0.32)	0.31** (0.23–0.39)	–	
16	Total Wellbeing	0.41** (0.32–0.48)	0.34** (0.25–0.42)	0.38** (0.29–0.46)	0.50** (0.43–0.57)	−0.47** (39–0.55)	0.52** (0.45–0.59)	0.36** (0.25–0.44)	−0.44** (−0.36-−0.51)	0.29** (0.20–0.39)	0.64** (0.58–0.70)	0.41** (0.32–0.49)	0.49** (0.41–0.57)	−0.18** (−**0.28 to**−**0.08)**	−0.33** (−**0.42 to**−**0.24)**	−0.41** (−**0.49 to**−**0.32)**	–
	**Sample**	766	766	765	768	769	770	767	769	764	732	761	762	747	732	769	754
	**Cronbach’s Alpha**	0.892	0.830	0.882	0.924	0.869	0.885	0.939	0.856	0.879	0.922	0.824	0.829	0.806	0.624	0.857	0.832
	**Mean**	17.48	12.40	13.58	13.28	13.06	17.38	15.99	13.63	14.15	72.97	25.39	43.07	1.48	0.38	25.85	94.41
	**AVE**	0.493	0.633	0.575	0.464	0.568	0.472	0.749	0.518	0.615	0.397	0.452	0.337	n/a	n/a	n/a	0.300
	**Skewness/Kurtosis**	−0.53/0.08	−0.63/0.66	−0.05/−0.67	−0.61/0.17	−0.59/−0.46	−0.56/0.06	−0.71/0.24	−0.84/0.85	−0.24/−0.55	−0.61/1.17	−0.09/0.46	0.10/−0.23	0.17/0.80	0.08/0.33	2.48/7.28	−0.06/0.33

Cells include r-value with 95% confidence-interval values in parentheses. *denotes p < 0.05 and **denotes p < 0.01. We used ^††^ to denote correlations that were statistically significant but with confidence intervals close to including zero. Gray font denotes no meaningful correlation. Black and bold indicates a significant correlation.

## Overview of Study 2

The aims of Study 2 were: first to test the revised items and factor structure from Study 1 with a previously unused sample (*n* = 270 from the original sample of 770); and second, to test the nomological network of the readiness scale, by examining their relations with indication of recent stress and demands, as well as protective traits such as resilience and self-control. Based on the theoretical rationale that resilience represents an array of traits that mitigate the effects of stress and demand on current readiness (e.g., Rutter, 1987; Connor and Davidson, 2003) we proposed that: (a) job demands, daily hassles, and perceived load in the most recent task would influence current readiness; (b) also influenced mitigating traits including resilience, stress mindset and self-control. We further predicted that (c) current readiness should be correlated to current affect, current distress, and a supervisor’s rating of current soldier readiness. Specifically, perceived readiness should be positively correlated with positive affect and supervisor’s ratings of soldier readiness, but negatively correlated with reported levels of distress and negative affect.

### Method – Study 2

Confirmatory Factor Analysis (CFA) was conducted on the remaining sample of 270 participants, randomly separated from the data used in Study 1. Subsequently, using the full 770 participants, correlational analysis was conducted using Pearson correlations, their 95% bootstrapped confidence intervals (CI), and average variance extracted (AVE) of the factors between: (a) ARMS factors; (b) recent sources of stress and demand (DISQ scores, Daily-Hassles and NASA-TLX); (c) resilience promoting traits (CD-RISC, Stress Mindset, Self-Control); (d) other current indicators of readiness including the K10 distress scale, PANAS affect score; and (e) a single item Visual Analog Scale rating of each soldier’s readiness by their current supervisor (see Tables 4, 5).

#### Measures

We selected a range of measures to assess convergent and discriminant validity of the newly developed ARMS. In the following paragraphs, we report the Cronbach alpha reliability from the original validation or a cited revalidation: for internal reliability scores in the current study see Tables 4, 5. To assess recent stress and load experienced by participants, we included three measures of recent stress: (a) chronic load using the 22-item Demand-Induced Strain Compensation Questionnaire to assess both cognitive and emotional occupational strain (Bova et al., 2015; α = 0.55–0.78); (b) recent low-level stresses using the 117-item Daily Hassles Scale (Kanner et al., 1981; Holm and Holroyd, 1992; α = 0.80–0.88); and (c) perceptions of how demanding the most recent task was – in terms of mental demand, physical demand, time-pressure, frustration and effort -using the NASA Task Load Index (NASA-TLX – Hart and Staveland, 1988; Xiao et al., 2005; α > 0.80).

To assess traits that protect performance capability from the effects of stress (i.e., resilience), we used: (a) the 25-item Connor-Davidson Resilience Scale (CD-RISC – Connor and Davidson, 2003; α = 0.89); (b) the 8-item Stress Mindset scale assessing the degree to which respondents view stress and challenge and developmental experiences versus detrimental (Crum et al., 2013; α = 0.86); (c) the 13-item Self-Control Scale (Tangney et al., 2004; α = 0.85); and (d) subjective wellbeing, which we hypothesized would be protective against stress and hassles in promoting acute readiness and capability. For this we used (Ryff and Keyes, 1995) 18-item psychological wellbeing scale (α = 0.33–0.56).

To assess other potential indices of acute readiness, we assessed: (a) affective state, using the Positive and Negative Affect Schedule (PANAS – Watson et al., 1988; α = 0.87–0.88); (b) 30-day distress using the Kessler-10-item Distress Scale (Sampasa-Kanyinga et al., 2018; α = 0.88); and (c) a rating by the supervisor, using a Visual Analog Scale (VAS) from 1 to 20 (1 = “not ready for likely duties,” 10 = “Suitably ready for likely duties”; 20 = “Beyond capable in relation to likely duties”). This VAS was developed specifically for this study. Participants gave additional consent for supervisor’s rating to be sought.

#### Analysis

Concurrent validity would be demonstrated by a significant correlation (*p* < 0.05 and confidence intervals not including 0) between corresponding factors (e.g., overall readiness and positive affect). Discriminant validity would be supported when two criteria were met: (1) 95% CI of the correlation between ARMS factors and the other factors did not include 1 or −1 (Bagozzi et al., 1991; Chan et al., 2018) and (2) *either* the Average Variance Extracted (AVE) of each factor was larger than their shared variance (i.e., square of correlation) with the other factors (Fornell and Larcker, 1981), *or* a visual inspection of the scatter plot between the two variables did not suggest any linear or curve-linear relationship.

### Results – Study 2

Using CFA, the model specified in Study 1 showed acceptable fit to the new data [χ^2^ (428) = 902.363, *p* < 0.001; χ^2^/428 = 2.1; CFI = 0.94; TLI = 0.93; SRMR = 0.06; RMSEA = 0.06 (90% CI: 0.06, 0.07)]. These are similar fit indices to those from Study 1, and broadly interpreted as on the boundary between “acceptable” and “good” model fit. Given the intended implementation by the stakeholders, we agreed that no further refinements were necessary to improve model-fit at this stage.

For concurrent validity, ARMS factors positively associated with each other, as well as positive affect, while negatively correlating with K10-distress and negative affect (see Table 4). For discriminant validity, the correlation 95% CI spanned zero between the ARMS factors and most aspects of the NASA-TLX, consistent with the notion that recent task-load alone is not a sufficient predictor of immediate readiness. Nevertheless, TLX-frustration demonstrated small correlation with ARMS subscales, suggesting that the emotional experience of frustration may be more relevant in determining perceptions of immediate readiness than other forms of task-load (see Table 4). Similarly, DISC-Q scores for emotional strain were negatively correlated to ARMS factors, although DISC-Q cognitive strain showed only small correlations. Thus, emotional load and emotional strain seem to be more promising indicators of readiness perceptions than recent cognitive load and/or strain. As expected - noting that fatigue items were reverse coded - all ARMS subscales were positively correlated with the resilience-promoting traits of resilience (CD-RISC), stress-mindset, self-control, and psychological wellbeing; while being negatively correlated with the total severity of recent hassles. ARMS subscales were positively correlated with positive affect, and negatively correlated with negative affect and K-10 distress, while meeting the criteria to demonstrate discriminant validity. The final Cronbach alpha for the overall ARMS scale was 0.949.

## Discussion

We developed and validated a psychometric instrument suitable for assessing acute readiness: the ARMS. The intent behind this tool was to facilitate rapid, reliable indications from personnel themselves of current individual and group capabilities for immediate tasks; as well as the ability to monitor how individuals and groups respond to training and deployment challenges. One data sample, conducted with Australian Army, was divided into two analyses, with findings supporting the key aspects of construct validity in this new psychometric tool.

### Summary of Findings

In Study 1, a total of 32 items for the ARMS received support in the forms of user-group endorsement, expert panel clarification, and then the demonstration of a factor structure that was largely consistent with expectations, including good model fit indices. The inclusion of four items to indicate “overall readiness” as a brief scale helps to facilitate the relatively un-intrusive monitoring of day-to-day readiness. The factor structure was kept simple, with no additional modeling beyond simply tallying the nine factors, with no sharing of error variances or additional modeling performed. In Study 2, the factor structure developed in Study 1 was supported, showing acceptable fit in a fresh sample. Further, the concurrent validity of the ARMS was evaluated by examining the correlations between subscales, as well as with targeted constructs such as recent task-load, time-of-day, affect, distress and supervisor-ratings of readiness. The subscales of the ARMS showed small-to-moderate intercorrelations as might be expected, and were also moderately associated with affect and K10-distress. The subscales “overall readiness” and “equipment readiness” showed small but statistically significant correlations to the supervisor’s rating of readiness. Most aspects of recent task-load did not associate to ARMS scores, although frustration in the most recent task was consistently correlated with ARMS scores. Additionally, ARMS subscales were largely correlated with recent stress, as indicated by severity of recent “daily hassles” and evaluations of occupational emotional demands (DISC-Q – De Jonge and Dormann, 2003). The initial validation of a psychometric tool for monitoring readiness across a range of contexts and job-roles provides the basis for further cross-sectional and longitudinal evaluations.

The main divergence from expectations was the emergence of separate “readiness” and “fatigue” factors under both the “physical” and “cognitive” subscales. While correlated, these items were not able to be modeled within single factors: i.e., they were not two ends of the same spectrum. A similar observation was made in a recent study of exercise readiness (Strohacker and Zakrajsek, 2016), with “freshness” and “fatigue” modeled as separate factors. Informal reviews with the user-group supported the interpretation that - while perhaps counter-intuitive - one can be fatigued by recent commitments and yet still “ready-to-go-again.” Likewise, one can feel *neither* fresh nor fatigued. As such a respondent could indicate physical or mental readiness-versus-fatigue to be any configuration of: (a) high:high; (b) high:low; (c) low:high or (d) low:low. In the studied population – perhaps faced by frequent combinations of physical and mental load, as well as certain cultural norms around admitting to weakness or vulnerability – it is possible that the observed pattern is unique to military: but that would largely support the need for further research to assess the suitability of the ARMS for different contexts. Upon returning to the wider literature, however, we did find examples of this pattern in other research. For example, Boolani and colleagues have characterized different correlates of both trait (Boolani and Manierre, 2019) and state fatigue-versus-energy (Boolani et al., 2018), as well as different responses to physical activity (Boolani et al., 2021). As such, our findings may be adding to a growing awareness that the experiences of energy and fatigue may be separate.

### Comparison to Previous Findings

We developed the ARMS as a highly useable and easily interpreted scale. In comparison to the bureaucratic-administrative exercise of monitoring training, equipment servicing and medical reporting processes (cf. Dabbieri, 2003), the ARMS represents a new and complimentary opportunity. This new psychometric instrument allows leaders/managers to quickly request validated, meaningful readiness scores from an individual, team or wider group: for example, in rapidly planning a new project, tender or crisis response. Not only would the resulting scores from the ARMS be almost immediate and in a consistent format, they would be provided by personnel with direct access to the relevant information. Further, information provided from the ARMS is readily integrated, synthesized and interpretable. These are all advancements complementing the existing management practices reliant on finding, studying and synthesizing information from a wide range of records: generated infrequently, and stored in different formats. Further, in comparison to the data from wearable technology that monitors physiological signs such as heart-rate variability, sleep or physical activity logs (cf. Domb, 2019; Seshadri et al., 2019), data from the ARMS should be more readily interpreted with minimal additional processing, and more readily collated at the group level to give information that planners and decision-makers may need. For example, information about lack of sleep or sustained physical exertion during operations may simply represent quite typical role requirements from long-distance travel or night-shift work, and so may tell the receiver little about the individual or group’s capabilities for further actions. Likewise, in a situation with limited connectivity and/or constraints on battery usage, the ARMS could be completed quickly using paper-and-pens, collated and interpreted *in situ*, without computational processing.

While previous psychometric instruments have been developed, the ARMS is arguably the only instrument to balance brevity (i.e., 4 core items + 28 expansion items) with capturing the multidimensional nature of acute readiness, across various roles and performance capabilities (cf. Bester and Stanz, 2007). Existing measures have either focused on specific aspects of readiness (e.g., cognitive readiness – Grier, 2012; exercise readiness – Strohacker and Zakrajsek, 2016; return from injury readiness – Conti et al., 2019) or broad overall appraisals of self-efficacy or anxiety. These measures have a much higher-ratio of items-per-component, representing a higher burden on respondents. Combining multiple different scales is also less suitable for immediate integration into a multidimensional model representing acute readiness. Similarly, psychometric measures of recent hassles, stress recovery, self-efficacy, affect, or anxiety are typically either: (a) too long; (b) not specific enough to be used and interpreted by users (i.e., broader academic concepts); (c) focused on past events, not the immediate here-and-now; or (d) some combination of a-c. The ARMS is the only instrument designed to be brief, readily interpreted by users themselves, and specifically focused on acute readiness.

### Next Steps

We recognize that the scale may require further validation in other contexts and groups in order to assess the generalizability. The sample in this study was, relatively young, well-educated and predominantly Caucasian, which may necessitate caution in seeking to apply these findings in other contexts/populations. Likewise, the emergence of physical and mental factors in “readiness” and “fatigue” warrants further examination in future research, although it is consistent with at least one previous study (Strohacker and Zakrajsek, 2016). Further, while the ARMS has been developed to be implemented into a regular program of monitoring, for example through an app or regular reporting practices, it has not been tested in that context. Hence, it would be important to determine how the instrument behaves when completed frequently, and whether there are ways of optimizing the utility of using the instrument - for example through carefully managing how often, what time-of-day, and after what events each subscale should be completed. This type of research would allow for the estimation of test-retest reliability, in circumstances where consistent scores would be expected, and also sensitivity to changes. While one aim of developing the ARMS was to facilitate a novel method for evaluating resilience – by observing acute psychological responses to challenges, adversity and training activities – the next logical step would be to evaluate the extent to which the instrument enables this evaluation. Future research assessing the use of the ARMS as an indicator of resilience is needed and would require repeated administration of the ARMS before and after a defined challenging event, or series of events.

### Limitations

The entire sample in this study included military personnel who were “on-base,” and so much less likely to be experiencing the fatigue generated by sustained operations and time away-from-home (or, for example, the fatigue caused by working from home over an extended period). Hence, it would be important to test the ARMS under diverse circumstances – both more civilian-relevant professions and also in more demanding military circumstances: potentially evolving items or factors to capture the effects of those activities. Finally, through the conduct of this study including talking to respondents and reviewing qualitative comments, coupled with the literature reviewed in preparing the ARMS, it is possible that there are other dimensions of readiness not assessed in the current measure. These forms might include social support from family and friends, rather than focusing on one’s unit or team, and also behavioral readiness such as having good routines throughout one’s day, or good diet and sleep patterns.

## Conclusion

Overall, therefore, the present study has developed and provided initial validation of an instrument to assess acute multidimensional readiness with a focus on performance capability. The resulting instrument offers many opportunities both to resolve or circumvent limitations in other measures, as well as opening up new avenues of research in further refining the scale, and optimizing the implementation into real-world settings. Furthermore, the development of the ARMS has advanced the conceptual understanding in this topic, by identifying a potentially important distinction between readiness as “freshness” versus “fatigue” as separate but correlated constructs. In addition to facilitating a new avenue of research on acute readiness itself - a previously under-researched concept – the development of the ARMS opens up opportunities to resolve real-world problems around readiness-monitoring, with implications for inferring resilience beyond cross-sectional trait measures, and potential to be adopted and applied in other contexts such as sport, occupational setting and health-monitoring. Finally, by offering the ability to capture subjective perceptions of readiness, the new scale also may facilitate research on how wearable technology and objectively measured indices relate to these subjective perceptions, and how this “nexus” of different forms of information might be useful in managing training, performance and recovery in a wide array of performance contexts. Overall, the ARMS makes it possible to assess acute readiness, for the group and individual, in a way that is both un-intrusive, easily interpreted and yet is psychometrically validated. As such, numerous new possibilities open up following the development of this scale.

## Data Availability Statement

The raw data supporting the conclusions of this article will be made available by the authors, without undue reservation.

## Ethics Statement

The studies involving human participants were reviewed and approved by Defense (Australia) Human Research Ethics Committee (Low Risk) University of Canberra Human Research Ethics Committee. The patients/participants provided their written informed consent to participate in this study.

## Author Contributions

RK conceptualizing the project alongside co-authors and stakeholders, collecting and analyzing data, and preparing the written report. AF, BR, and MS provided important conceptual and methodological inputs in designing, implementing and interpreting the study (BR also participated in data collection). TN and MW are statisticians who provided critical oversight in planning, implementing and interpreting the analysis of data. LM was the Army liaison who ensured access to data collection opportunities and oversaw the secure collection and storage of data, as well as assisting in design and interpretation phases of the project. DC was the lead contributor from Defense Science Technology Group, playing a crucial role in the design, planning and facilitation of the study: spanning conceptualization, ethical approvals, methods and the planning of data collection. All authors contributed to the article and approved the submitted version.

## Conflict of Interest

The authors declare that the research was conducted in the absence of any commercial or financial relationships that could be construed as a potential conflict of interest.

## Publisher’s Note

All claims expressed in this article are solely those of the authors and do not necessarily represent those of their affiliated organizations, or those of the publisher, the editors and the reviewers. Any product that may be evaluated in this article, or claim that may be made by its manufacturer, is not guaranteed or endorsed by the publisher.

## Footnotes

1 The Australian Army, like many other armed forces, enacts a training cycle (the “Force Generation Cycle”): rotating personnel through three phases of Ready, Readying, and Reset. Each phase comes with different expectations, activities, and support requirements (Australian Army, 2014). While adopting this cyclical approach facilitates a sound balance of training, recovery and active duties, the Australian Army subsequently identified an opportunity to optimize and individualize activities within this cycle (Gilmore, 2016) – which precipitated the current research project.

## References

[B1] Adams B. D. Hall C. D. T. Thomson M. H. (2009). *Military Individual Readiness. État de Préparation Militaire de L’individu (DRDC No. CR-2009-075).* Ottawa, ON: Department of National Defence.

[B2] Agaibi C. E. Wilson J. P. (2005). Trauma, PTSD, and resilience: a review of the literature. *Trauma Viol. Abuse* 6 195–216. 10.1177/1524838005277438 16237155

[B3] American Educational Research Association [AERA], American Psychological Association [APA], and National Council on Measurement in Education [NCME] (2014). *Standards for Educational and Psychological Testing.* Washington, DC: American Educational Research Association.

[B4] Anthoine E. Moret L. Regnault A. Sbille V. Hardouin J. B. (2014). Sample size used to validate a scale: a review of publications on newly-developed patient reported outcomes measures. *Health Q. Life Outcomes* 12 1–10. 10.1186/s12955-014-0176-2 25492701 PMC4275948

[B5] Apiecionek L. Kosowski T. Kruszynski H. Piotrowski M. Palka R. (2012). “The concept of integration tool for the civil and military service cooperation during emergency response operations,” in *Proceedings of the 2012 Military Communications and Information Systems Conference, MCC 2012*, New York, NY.

[B6] Army Regulation 220-1 (2003). *Unit Status Reporting.* Washington, DC: U.S. Army.

[B7] Asparouhov T. Muthén B. (2009). Exploratory structural equation modeling. *Struct. Equ. Model.* 16 397–438. 10.1080/10705510903008204

[B8] Australian Army (2014). *Chief of Army Opening Address to Land Forces 2014, Army, September 24, 2014.* Available online at: https://www.army.gov.au/our-work/speeches-and-transcripts/chief-of-army-opening-address-to-land-forces-2014 (accessed February 6, 2018).

[B9] Australian Bureau of Statistics (2016). *2016 Census QuickStats.* Canberra: Australian National Government.

[B10] Bagozzi R. P. Yi Y. Phillips L. W. (1991). Assessing construct validity in organizational research. *Admin. Sci. Q.* 36 421–458. 10.2307/2393203

[B11] Baig M. M. Gholam-Hosseini H. Moqeem A. A. Mirza F. Lindén M. (2017). A systematic review of wearable patient monitoring systems – current challenges and opportunities for clinical adoption. *J. Med. Syst.* 7 109–115. 10.1007/s10916-017-0760-1 28631139

[B12] Bakker A. B. Demerouti E. (2007). The job demands-resources model: state of the art. *J. Manag. Psychol.* 22 309–328. 10.1108/02683940710733115

[B13] Bakker A. B. Demerouti E. Euwema M. C. (2005). Job resources buffer the impact of job demands on burnout. *J. Occupat. Health Psychol.* 10 170–180. 10.1037/1076-8998.10.2.170 15826226

[B14] Bakker A. B. Hakanen J. J. Demerouti E. Xanthopoulou D. (2007). Job resources boost work engagement, particularly when job demands are high. *J. Educ. Psychol.* 99 274–284. 10.1037/0022-0663.99.2.274

[B15] Bakker A. B. van Veldhoven M. Xanthopoulou D. (2010). Beyond the demand-control model: thriving on high job demands and resources. *J. Pers. Psychol.* 9 3–16. 10.1027/1866-5888/a000006

[B16] Bandalos D. L. (2014). Relative performance of categorical diagonally weighted least squares and robust maximum likelihood estimation. *Struct. Equ. Model.* 21 102–116. 10.1080/10705511.2014.859510

[B17] Bandodkar A. J. Wang J. (2014). Non-invasive wearable electrochemical sensors: a review. *Trends Biotechnol.* 32 363–371. 10.1016/j.tibtech.2014.04.005 24853270

[B18] Bandura A. (1977). Self-efficacy: toward a unifying theory of behavioral change. *Psychol. Rev.* 84 191–215. 10.1037/0033-295X.84.2.191 847061

[B19] Bartholomew K. J. Ntoumanis N. Ryan R. M. Thøgersen-Ntoumani C. (2011). Psychological need thwarting in the sport context: assessing the darker side of athletic experience. *J. Sport Exerc. Psychol.* 33 75–102. 10.1123/jsep.33.1.75 21451172

[B20] Bester P. C. Stanz K. J. (2007). The conceptualisation and measurement of combat readiness for peace-support operations – an exploratory study. *SA J. Industr. Psychol.* 33 68–78. 10.4102/sajip.v33i3.398

[B21] Bhavsar N. Bartholomew K. J. Quested E. Gucciardi D. F. Thøgersen-Ntoumani C. Reevee J. (2020). Measuring psychological need states in sport: theoretical considerations and a new measure. *Psychol. Sport Exerc.* 47:101617. 10.1016/j.psychsport.2019.101617

[B22] Blackburn D. (2014). Military individual readiness: an overview of the individual components of the adam, hall, and Thomson model adapted to the Canadian armed forces. *Can. Milit. J.* 14 36–45.

[B23] Blair M. R. Body S. F. Croft H. G. (2017). Relationship between physical metrics and game success with elite rugby sevens players. *Intern. J. Perform. Analys. Sport* 15 1037–1046. 10.1080/24748668.2017.1348060

[B24] Boolani A. Bahr B. Milani I. Caswell S. Cortes N. Smith M. L. (2021). Physical activity is not associated with feelings of mental energy and fatigue after being sedentary for 8 hours or more. *Ment. Health Phys. Activ.* 21:100418. 10.1016/j.mhpa.2021.100418

[B25] Boolani A. Manierre M. (2019). An exploratory multivariate study examining correlates of trait mental and physical fatigue and energy. *Fatigue Biomed. Health Behav.* 7 29–40. 10.1080/21641846.2019.1573790

[B26] Boolani A. O’Connor P. J. Reid J. Ma S. Mondal S. (2018). Predictors of feelings of energy differ from predictors of fatigue. *Fatigue Biomed. Health Behav.* 7 12–28. 10.1080/21641846.2018.1558733

[B27] Bova N. De Jonge J. Guglielmi D. (2015). The demand-induced strain compensation questionnaire: a cross-national validation study. *Stress Health* 31 236–244. 10.1002/smi.2550 26252420

[B28] Burr R. L. G. (2018). *Accelerated Warfare: Futures Statement for An Army in Motion.* Canberra: Australian Army.

[B29] Byers J. C. Bittner A. C. Hill S. G. (1989). “Traditional and raw task load index (TLX) correlations: are paired comparisons necessary?,” in *Advances in Industrial Ergonomics and Safety*, Vol. 32 ed. Mital A. (London: Taylor Francis), 481–485.

[B30] Cattell R. B. (1966). The screen test for the number of factors. *Multivariate Behav. Res.* 1, 245–276. 10.1207/s15327906mbr0102_10 26828106

[B31] Chan D. K. C. Keegan R. J. Lee A. S. Y. Yang S. X. Zhang L. Rhodes R. E. (2018). Towards a better assessment of perceived social influence: the relative role of significant others on young athletes. *Scand. J. Med. Sci. Sports* 29 286–298. 10.1111/sms.13320 30320928

[B32] Chan E. K. H. (2014). “Standards and guidelines for validation practices: development and evaluation of measurement instruments,” in *Validity and Validation in Social, Behavioral, and Health Sciences*, eds Zumbo B. D. Chan E. K. H. (New York, NY: Springer), 9–24.

[B33] Chesney M. A. Neilands T. B. Chambers D. B. Taylor J. M. Folkman S. (2006). A validity and reliability study of the coping self-efficacy scale. *Br. J. Health Psychol.* 11 421–437. 10.1348/135910705X53155 16870053 PMC1602207

[B34] Chuang C. H. Jackson S. E. Jiang Y. (2016). Can knowledge-intensive teamwork be managed? examining the roles of HRM systems, leadership, and tacit knowledge. *J. Manag.* 42 524–554. 10.1177/0149206313478189

[B35] Comrey A. L. Lee H. B. (1992). *A First Course in Factor Analysis*, 2nd Edn, Hillsdale, NJ: Lawrence Erlbaum.

[B36] Connor K. M. Davidson J. R. T. (2003). Development of a new resilience scale: the Connor-Davidson Resilience scale (CD-RISC). *Depress. Anxiety* 18 76–82. 10.1002/da.10113 12964174

[B37] Conti C. di Fronso S. Pivetti M. Robazza C. Podlog L. Bertollo M. (2019). Well-come back! Professional basketball players perceptions of psychosocial and behavioral factors influencing a return to pre-injury levels. *Front. Psychol.* 10:222. 10.3389/fpsyg.2019.00222 30800089 PMC6375854

[B38] Coyne J. O. C. Gregory Haff G. Coutts A. J. Newton R. U. Nimphius S. (2018). The current state of subjective training load monitoring—A practical perspective and call to action. *Sports Med. Open* 4:58. 10.1186/s40798-018-0172-x 30570718 PMC6301906

[B39] Crane M. F. (2017). *Managing for Resilience: A Practical Guide for Employee Wellbeing and Organizational Performance.* London: Routledge.

[B40] Crawford J. R. Henry J. D. (2010). The Positive and Negative Affect Schedule (PANAS): construct validity, measurement properties and normative data in a large non-clinical sample. *Br. J. Clin. Psychol.* 43 245–265. 10.1348/0144665031752934 15333231

[B41] Cronbach L. J. (1951). Coefficient alpha and the internal structure of tests. *Psychometrika* 16 297–334. 10.1007/BF02310555

[B42] Crum A. J. Salovey P. Achor S. (2013). Rethinking stress: the role of mindsets in determining the stress response. *J. Pers. Soc. Psychol.* 104 716–733. 10.1037/a0031201 23437923

[B43] Dabbieri R. (2003). Readiness: it’s everybody’s business. *Engineer* 21 28–33.

[B44] De Jonge J. Dormann C. (2003). “The DISC model: demand-induced strain compensation mechanisms in job stress,” in *Occupational Stress in the Service Professions*, eds Dollard M. F. Winefield A. H. Winefield H. R. (London: Taylor and Francis), 43–74.

[B45] Demerouti E. Nachreiner F. Bakker A. B. Schaufeli W. B. (2001). The job demands-resources model of burnout. *J. Appl. Psychol.* 86 499–512. 10.1037/0021-9010.86.3.499 11419809

[B46] Der-Martirosian C. Radcliff T. A. Gable A. R. Riopelle D. Hagigi F. A. Brewster P. (2017). Assessing hospital disaster readiness over time at the US department of veterans affairs. *Prehosp. Disaster Med.* 32 46–57. 10.1017/S1049023X16001266 27964767

[B47] Dien J. (2010). Evaluating two-step PCA of ERP data with geomin, infomax, oblimin, promax, and varimax rotations. *J. Psychophysiol.* 47, 170–183. 10.1111/j.1469-8986.2009.00885.x 19761521

[B48] Domb M. (2019). “Wearable devices and their Implementation in various domains,” in *Wearable Devices - the Big Wave of Innovation*, ed. Nasiri N. (London: IntechOpen), 10.5772/intechopen.86066

[B49] Elfering A. Grebner S. Leitner M. Hirschmüller A. Kubosch E. J. Baur H. (2017). Quantitative work demands, emotional demands, and cognitive stress symptoms in surgery nurses. *Psychol. Health Med.* 22 604–610. 10.1080/13548506.2016.1200731 27326467

[B50] Evenson K. R. Goto M. M. Furberg R. D. (2015). Systematic review of the validity and reliability of consumer-wearable activity trackers. *Intern. J. Behav. Nutr. Phys. Activity* 12:159. 10.1186/s12966-015-0314-1 26684758 PMC4683756

[B51] Faber A. J. Willerton E. Clymer S. R. MacDermid S. M. Weiss H. M. (2008). Ambiguous absence, ambiguous presence: a qualitative study of military reserve families in wartime. *J. Fam. Psychol.* 22 222–230. 10.1037/0893-3200.22.2.222 18410209

[B52] Forces Command Resilience Plan (2015). *Headquarters Forces Command Resilience Report Financial Year 2017/2018.* Available online at: https://cove.army.gov.au/sites/default/files/06-07/06/180227-Guide-HQ-FORCOMD-Military-Health-and-Psychology-Commanding-Office.pdf (accessed July 2018).

[B53] Fornell C. Larcker D. F. (1981). Evaluating structural equation models with unobservable variables and measurement error. *J. Market. Res.* 18 39–50. 10.2307/3151312

[B54] Gilmore P. W. (2016). *Leading a Resilient Force. Army Research Paper, No. 11.* Available online at: https://researchcentre.army.gov.au/sites/default/files/161107_gilmore_resilient.pdf (accessed July, 2018).

[B55] Grier R. A. (2012). Military cognitive readiness at the operational and strategic levels: a theoretical model for measurement development. *J. Cogn. Eng. Decis. Mak.* 6 358–392. 10.1177/1555343412444606

[B56] Grier R. A. Fletcher J. D. Morrison J. E. (2012). Defining and measuring military cognitive readiness. *Proc. Hum. Fact. Ergon. Soc.* 56 435–437. 10.1177/1071181312561098

[B57] Griffith J. (2006). What do the soldiers say: needed ingredients for determining unit readiness. *Arm.Forc. Soc.* 32 367–388. 10.1177/0095327X05280555

[B58] Hart S. G. Staveland L. E. (1988). “Development of NASA-TLX (Task Load Index): results of empirical and theoretical research,” in *Human Mental Workload*, eds Hancock P. A. Meshkati N. (Amsterdam: North Holland Press), 10.1016/S0166-4115(08)62386-9

[B59] Hellewell S. C. Cernak I. (2018). Measuring resilience to operational stress in canadian armed forces personnel. *J. Traum. Stress* 31 89–101. 10.1002/jts.22261 29465774

[B60] Holm J. E. Holroyd K. A. (1992). The daily hassles scale (revised): does it measure stress or symptoms? *Behav. Assess.* 14 465–482.

[B61] Hooper D. Coughlan J. Mullen M. R. (2008). Structural equation modelling: guidelines for determining model fit. *Electron. J. Bus. Res. Methods* 6 53–60. 10.12691/amp-5-3-2

[B62] Hu L. T. Bentler P. M. (1999). Cutoff criteria for fit indexes in covariance structure analysis: conventional criteria versus new alternatives. *Struct. Equ. Model.* 6 1–55. 10.1080/10705519909540118

[B63] IBM Corp (2016). *IBM SPSS Statistics for Windows, Version 24.0.* Armonk, NY: IBM Corp.

[B64] Kanner A. D. Coyne J. C. Schaefer C. Lazarus R. S. (1981). Comparison of two modes of stress measurement: daily hassles and uplifts versus major life events. *J. Behav. Med.* 4 1–40. 10.1007/BF00844845 7288876

[B65] Kölling S. Hitzschke B. Holst T. Ferrauti A. Meyer T. Pfeiffer M. (2015). Validity of the acute recovery and stress scale: training monitoring of the German Junior National Field Hockey Team. *Intern. J. Sports Sci. Coach.* 10 529–542. 10.1260/1747-9541.10.2-3.529 29106335

[B66] Kuiper R. A. Pesut D. J. (2004). Promoting cognitive and metacognitive reflective reasoning skills in nursing practice: self-regulated learning theory. *J. Adv. Nurs*. 45 381–391. 10.1046/j.1365-2648.2003.02921.x 14756832

[B67] Lane J. Lane A. M. Kyprianou A. (2004). Self-efficacy, self-esteem and their impact on academic performance. *Soc. Behav. Pers.* 32 247–256. 10.2224/sbp.2004.32.3.247

[B68] Lee H. H. Cranford J. A. (2008). Does resilience moderate the associations between parental problem drinking and adolescents’ internalizing and externalizing behaviors? A study of Korean adolescents. *Drug Alcoh. Depend.* 96 213–221. 10.1016/j.drugalcdep.2008.03.007 18440164

[B69] Lee J. Wheeler K. James D. A. (2018). *Wearable Sensors in Sport A Practical Guide to Usage and Implementation (Springer Briefs in Applied Sciences and Technology)*, 1st Edn, London: Springer.

[B70] Lewig K. A. Xanthopoulou D. Bakker A. B. Dollard M. F. Metzer J. C. (2007). Burnout and connectedness among Australian volunteers: a test of the job demands–resources model. *J. Vocat. Behav.* 71, 429–445. 10.1016/j.jvb.2007.07.003

[B71] Luthar S. S. Cicchetti D. Becker B. (2000). The construct of resilience: a critical evaluation and guidelines for future work. *Child Dev.* 71 543–562. 10953923 10.1111/1467-8624.00164 PMC1885202

[B72] Marsh H. W. Hau K. T. Wen Z. (2004). In search of golden rules: comment on hypothesis-testing approaches to setting cutoff values for fit indexes and dangers in overgeneralizing Hu and Bentler’s (1999) findings. *Struct. Equ. Model.* 11 320–341. 10.1207/s15328007sem1103_2

[B73] McFarland R. G. Rode J. C. Shervani T. A. (2016). A contingency model of emotional intelligence in professional selling. *J. Acad. Mark. Sci.* 44 108–118.

[B74] Muthén L. Muthén B. (2012). *Mplus Version 7 User’s Guide.* Los Angeles, CA: Muthén & Muthén, 10.1111/j.1532-5415.2004.52225.x

[B75] Nahrgang J. D. Morgeson F. P. Hofmann D. A. (2011). Safety at work: a meta-analytic investigation of the link between job demands, job resources, burnout, engagement, and safety outcomes. *J. Appl. Psychol.* 96 71–94. 10.1037/a0021484 21171732

[B76] Nässi A. Ferrauti A. Meyer T. Pfeiffer M. Kellmann M. (2017). Psychological tools used for monitoring training responses of athletes. *Perform. Enhanc. Health* 5 125–133. 10.1016/j.peh.2017.05.001

[B77] Netemeyer R. G. Bearden W. O. Sharma S. (2003). *Scaling Procedures.* Thousand Oaks, CA: SAGE Publications, Inc. 10.4135/9781412985772

[B78] Netemeyer R. Bearden W. Sharma S. (2012). “Validity,” in *Scaling Procedures*, eds Netemeyer R. G. Bearden W. O. Sharma S. (Thousand Oaks, CA: Sage Publications), 72–88. 10.4135/9781412985772.n4

[B79] Ng J. Y. Lonsdale C. Hodge K. (2011). The basic needs satisfaction in sport scale (BNSSS): instrument development and initial validity evidence. *Psychol. Sport Exerc.* 12 257–264. 10.1016/j.psychsport.2010.10.006 18648109

[B80] Nunnally J. C. (1978). *Psychometric Theory*, 2nd Edn, New York, NY: McGraw-Hill.

[B81] Pangallo A. Zibarras L. Lewis R. Flaxman P. (2015). Resilience through the lens of interactionism: a systematic review. *Psychol. Assess.* 27 1–20. 10.1037/pas0000024 25222438

[B82] Peake J. M. Kerr G. Sullivan J. P. (2018). A critical review of consumer wearables, mobile applications, and equipment for providing biofeedback, monitoring stress, and sleep in physically active populations. *Front. Physiol.* 9:743. 10.3389/fphys.2018.00743 30002629 PMC6031746

[B83] Peterson C. Park N. Castro C. A. (2011). Assessment for the U.S. Army comprehensive soldier fitness program: the global assessment tool. *Am. Psychol.* 66 10–18. 10.1037/a0021658 21219043

[B84] Preston C. C. Colman A. M. (2000). Optimal number of response categories in rating scales: reliability, validity, discriminating power, and respondent preferences. *Acta Psychol.* 104 1–15. 10.1016/S0001-6918(99)00050-5 10769936

[B85] Reivich K. J. Seligman M. E. P. McBride S. (2011). Master resilience training in the U.S. Army. *Am. Psychol.* 66 25–34. 10.1037/a0021897 21219045

[B86] Rhemtulla M. Brosseau-Liard P. É Savalei V. (2012). When can categorical variables be treated as continuous? A comparison of robust continuous and categorical SEM estimation methods under suboptimal conditions. *Psychol. Methods* 17, 354–373. 10.1037/a0029315 22799625

[B87] Richardson G. E. (2002). The metatheory of resilience and resiliency. *J. Clin. Psychol.* 58, 307–321. 10.1002/jclp.10020 11836712

[B88] Robertson I. T. Cooper C. L. Sarkar M. Curran T. (2015). Resilience training in the workplace from 2003 to 2014: a systematic review. *J. Occup. Organ. Psychol.* 88 533–562. 10.1111/joop.12120

[B89] Rocchi M. Pelletier L. Cheung S. Baxter D. Beaudry S. (2017). Assessing need-supportive and need-thwarting interpersonal behaviours: the Interpersonal Behaviours Questionnaire (IBQ). *Pers. Individ. Differ.* 104, 423–433. 10.1016/j.paid.2016.08.034

[B90] Rollins J. W. (2001). Civil-military cooperation (CIMIC) in crisis response operations: the implications for NATO. *Intern. J. Phytoremed.* 8 122–129. 10.1080/13533310108413883

[B91] Roy T. C. Lopez H. P. (2013). A comparison of deployed occupational tasks performed by different types of military battalions and resulting low back pain. *Milit. Med.* 178:e0937-43. 10.7205/milmed-d-12-00539 23929059

[B92] Rutherford P. (2013). Training in the army: meeting the needs of a changing culture. *Train. Dev.* 40 8–10.

[B93] Rutter M. (1987). Psychosocial resilience and protective mechanisms. *Am. J. Orthopsych.* 57 316–331. 10.1111/j.1939-0025.1987.tb03541.x 3303954

[B94] Ryckman R. M. Robbins M. A. Thornton B. Cantrell P. (1982). Development and validation of a physical self-efficacy scale. *J. Pers. Soc. Psychol.* 42 891–900. 10.1037/0022-3514.42.5.891

[B95] Ryff C. D. (1989). Happiness is everything, or is it? Explorations on the meaning of psychological well-being. *J. Pers. Soc. Psychol.* 57 1069–1081. 10.1037/0022-3514.57.6.1069

[B96] Ryff C. D. Keyes C. L. M. (1995). The structure of psychological well-being revisited. *J. Pers. Soc. Psychol.* 69 719–727. 10.1037/0022-3514.69.4.719 7473027

[B97] Sampasa-Kanyinga H. Zamorski M. A. Colman I. (2018). The psychometric properties of the 10-item Kessler psychological distress scale (K10) in Canadian military personnel. *PLoS One* 13:e0196562. 10.1371/journal.pone.0196562 29698459 PMC5919406

[B98] Seshadri D. R. Li R. T. Voos J. E. Rowbottom J. R. Alfes C. M. Zorman C. A. (2019). Wearable sensors for monitoring the physiological and biochemical profile of the athlete. *NPJ Digit. Med.* 72 2–16. 10.1038/s41746-019-0150-9 31341957 PMC6646404

[B99] Shah N. Irani Z. Sharif A. M. (2017). Big data in an HR context: exploring organizational change readiness, employee attitudes and behaviors. *J. Bus. Res.* 70 366–378. 10.1016/j.jbusres.2016.08.010

[B100] Sharma N. Herrnschmidt J. Claes V. Bachnick S. De Geest S. Simon M. (2018). Organizational readiness for implementing change in acute care hospitals: an analysis of a cross-sectional, multicentre study. *J. Adv. Nurs.* 74 2798–2808. 10.1111/jan.13801 30019540

[B101] Sherer M. Maddux J. E. Mercandante B. Prentice-Dunn S. Jacobs B. Rogers R. W. (1982). The self-efficacy scale: construction and validation. *Psychol. Rep.* 51 663–671. 10.2466/pr0.1982.51.2.663

[B102] Strohacker K. Keegan R. J. Beaumont C. T. Zakrajsek R. A. (2021). Applying P-technique factor analysis to explore person-specific models of readiness-to-exercise. *Front. Sports Act. Living* 3:685813. 10.3389/fspor.2021.685813 34250469 PMC8267010

[B103] Strohacker K. Zakrajsek R. A. (2016). Determining dimensionality of exercise readiness using exploratory factor analysis. *J. Sports Sci. Med.* 15 229–238. 27274659 PMC4879435

[B104] Tangney J. P. Baumeister R. F. Boone A. L. (2004). High self-control predicts good adjustment, less pathology, better grades, and interpersonal success. *J. Pers.* 72 271–324. 10.1111/j.0022-3506.2004.00263.x 15016066

[B105] Thompson B. (2004). *Exploratory and Confirmatory Factor Analysis: Understanding Concepts and Applications.* Washington, DC: American Psychological Association, 10.1037/10694-000

[B106] Training and Doctrine Command (2011). *The U.S. Army Learning Concept for 2015. TRADOC Pamphlet 525-8-2.* Vilnius: Training and Doctrine Command.

[B107] Van Dierendonck D. (2005). The construct validity of Ryff’s scales of psychological well-being and its extension with spiritual well-being. *Pers. Individ. Differ.* 36 629–643. 10.1007/BF03071043

[B108] Watson D. Clark L. A. Tellegen A. (1988). Development and validation of brief measures of positive and negative affect: the PANAS scales. *J. Pers. Soc. Psychol.* 54 1063–1070. 10.1037/0022-3514.54.6.1063 3397865

[B109] Wen K. F. Nor N. M. Soon L. L. (2014). “A survey on the measure of combat readiness,” in *Proceedings of the Statistics and Operational Research International Conference (SORIC): AIP Conference Proceedings*, Vol. 1613 London.

[B110] Wetzel A. P. (2012). Factor analysis methods and validity evidence: a review of instrument development across the medical education continuum. *Acad. Med.* 87 1060–1069. 10.1097/ACM.0b013e31825d305d 22722361

[B111] Windle G. Bennett K. M. Noyes J. (2011). A methodological review of resilience measurement scales. *Health Q. Life Outcomes* 9:8. 10.1186/1477-7525-9-8 21294858 PMC3042897

[B112] Xiao Y. M. Wang Z. M. Wang M. Z. Lan Y. J. Z. (2005). The appraisal of reliability and validity of subjective workload assessment technique and NASA-task load index. *Chin. J. Indust. Hyg. Occup. Dis.* 23 178–181. 16124892

[B113] Yip B. Rowlinson S. (2006). Coping strategies among construction professionals: cognitive and behavioural efforts to manage job stressors. *J. Educ. Built Environ.* 1 70–79.

