# Supplementary figures and images for: Development and Initial Validation of an Acute Readiness Monitoring Scale in Military Personnel
Source: Front Psychol. 2021 Nov 18;12:738609. doi: 10.3389/fpsyg.2021.738609 (PMC8636321; doi:10.3389/fpsyg.2021.738609)

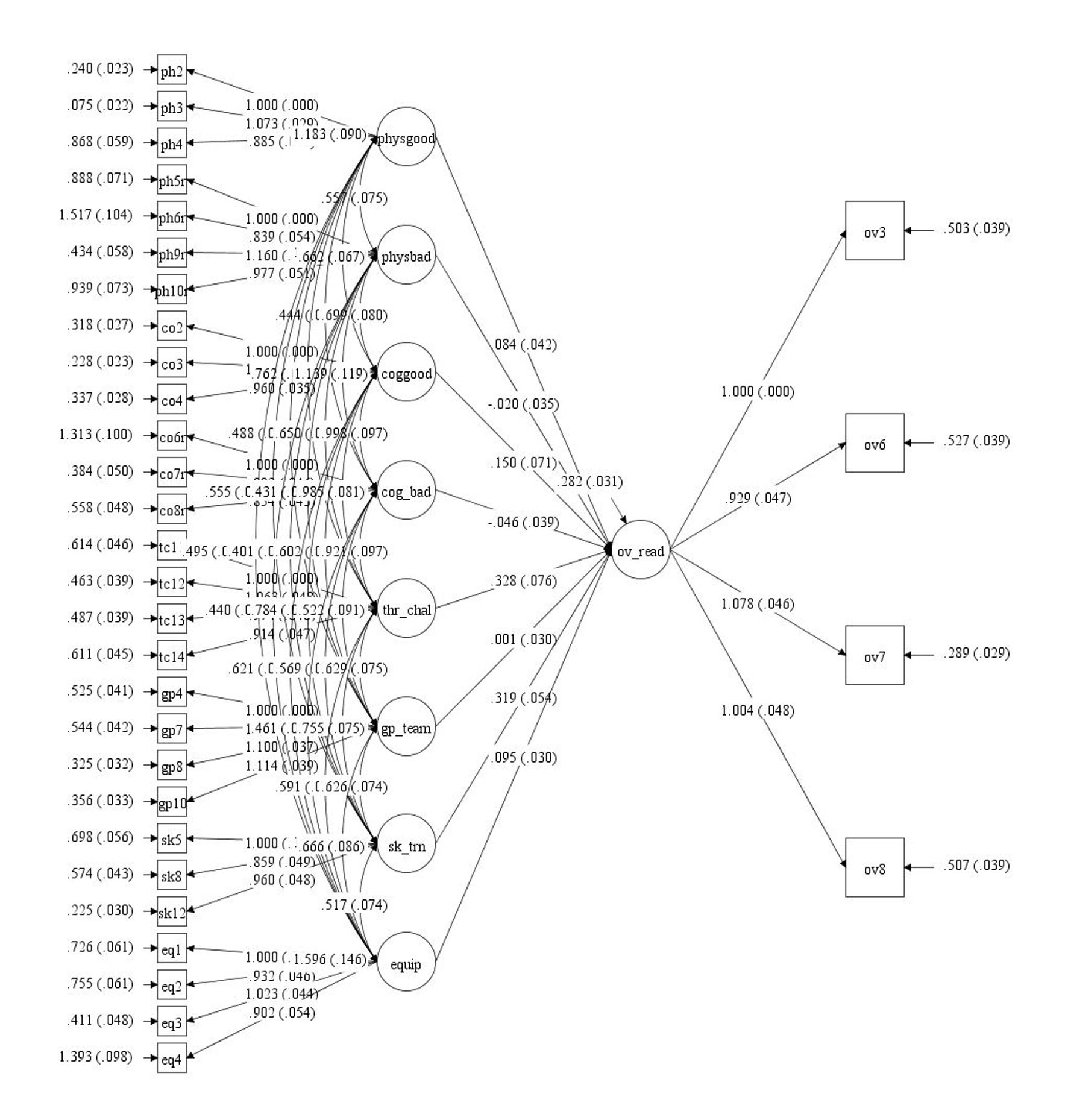

Supplement: Supplementary file 1 [file Image_1.JPEG]
