# Supplementary material for: Development and Initial Validation of an Acute Readiness Monitoring Scale in Military Personnel
Source: Front Psychol. 2021 Nov 18;12:738609. doi: 10.3389/fpsyg.2021.738609 (PMC8636321; doi:10.3389/fpsyg.2021.738609)
Supplement: Supplementary file 2 [file Data_Sheet_1.docx]

Examples of code

**BASIC EFA CODE – starting with all items**

Title: ReadyEFA;

Data:

File is C:\Users\s428940\MPlus Files/REA_EFA1.dat ;

Variable:

Names are OV1 OV2 OV3 OV4 OV5 OV6

OV7 OV8 OV9 OV10 OV11 OV12

PH1 PH2 PH3 PH4 PH5R PH6R

PH7 PH8 PH9R PH10R PH11R PH12R

CO1 CO2 CO3 CO4 CO5 CO6R

CO7R CO8R CO9 CO10 CO11R CO12R

TC1 TC2 TC3R TC4R TC5 TC6R

TC7R TC8 TC9R TC10 TC11 TC12

TC13 TC14 GP1 GP2 GP3 GP4

GP5 GP6 GP7 GP8 GP9 GP10

GP11 GP12 SK1 SK2 SK3 SK4

SK5 SK6R SK7R SK8 SK9 SK10

SK11 SK12 EQ1 EQ2 EQ3 EQ4

EQ5R EQ6 EQ7 EQ8 EQ9 EQ10R

EQ11R EQ12R EQ13 EQ14 ;

Missing are . ;

USEVARIABLES are

OV3 OV6

OV7 OV8

PH2 PH3 PH4

PH5R PH6R PH9R PH10R

CO2 CO3 CO4 CO6R

CO7R CO8R

TC11 TC12

TC13 TC14

GP4

GP7 GP8 GP10

SK5 SK8 SK12

EQ1 EQ2 EQ3 EQ4

;

! This approach uses ESEM (Exploratory Structural Equation Modeling) and allow

**First pass of EFA – developing ‘overall’ scale**

Title: ReadyEFA;

Data:

File is C:\Users\s428940\MPlus Files/REA_EFA1.dat ;

Variable:

Names are OV1 OV2 OV3 OV4 OV5 OV6

OV7 OV8 OV9 OV10 OV11 OV12

PH1 PH2 PH3 PH4 PH5R PH6R

PH7 PH8 PH9R PH10R PH11R PH12R

CO1 CO2 CO3 CO4 CO5 CO6R

CO7R CO8R CO9 CO10 CO11R CO12R

TC1 TC2 TC3R TC4R TC5 TC6R

TC7R TC8 TC9R TC10 TC11 TC12

TC13 TC14 GP1 GP2 GP3 GP4

GP5 GP6 GP7 GP8 GP9 GP10

GP11 GP12 SK1 SK2 SK3 SK4

SK5 SK6R SK7R SK8 SK9 SK10

SK11 SK12 EQ1 EQ2 EQ3 EQ4

EQ5R EQ6 EQ7 EQ8 EQ9 EQ10R

EQ11R EQ12R EQ13 EQ14 ;

Missing are . ;

USEVARIABLES are

OV1 OV2 OV3 OV4 OV5 OV6

OV7 OV8 OV9 OV10 OV11 OV12

;

! This is EFA (Exploratory Factor Analysis) with 3 factors;

ANALYSIS: TYPE=EFA 1 5;

ESTIMATOR=ML;

! ROTATION = VARIMAX; ! Default rotation is Oblique;

!PARALLEL=100; ! Parallel Analysis(100=#of samples used)

!REPSE=BOOTSTRAP;

****FOR EFA - ****

ANALYSIS: ESTIMATOR=ML;

! ROTATION = VARIMAX;

****FOR COMPARING FACTOR SOLUTIONS****

ANALYSIS: TYPE=EFA 9 10; ****ALLOWS COMPARISON OF DIFFERENT NUMBERS OF FACTORS****

ESTIMATOR=ML;

! ROTATION = VARIMAX; ! **Default rotation is Oblique;**

!PARALLEL=100; ! Parallel Analysis(100=#of samples used)

!REPSE=BOOTSTRAP;

****ESEM STEP ALLOWS EVALUATION OF EMERGING MODEL AND SUGGESTS MODIFICATIONS****

MODEL: OV_READ BY OV3 OV6 OV7 OV8 ;

PHYSGOOD BY PH2 PH3 PH4 ;

PHYSBAD BY PH5R PH6R PH9R PH10R ;

COGGOOD BY CO2 CO3 CO4 ;

COG_BAD BY CO6R CO7R CO8R ;

THR_CHAL BY TC11 TC12 TC13 TC14 ;

GP_TEAM BY GP4 GP7 GP8 GP10 ;

SK_TRN BY SK5 SK8 SK12 ;

EQUIP BY EQ1 EQ2 EQ3 EQ4 ;

OV_READ ON PHYSGOOD PHYSBAD COGGOOD

COG_BAD THR_CHAL GP_TEAM SK_TRN

EQUIP ;

OUTPUT: STDYX MODINDICES(ALL) ; !

***CFA- using second tranche of data****

Title: ReadyCFA_PRACTICE;

Data:

File is C:\Users\s428940\MPlus Files/CFA_DATA_READI.dat ;

Variable:

Names are OV1 OV2 OV3 OV4 OV5 OV6 OV7 OV8 OV9 OV10 OV11 OV12 PH1 PH2 PH3 PH4 PH5R PH6R PH7 PH8 PH9R PH10R PH11R PH12R CO1 CO2 CO3 CO4 CO5 CO6R CO7R CO8R CO9 CO10 CO11R CO12R TC1 TC2 TC3R TC4R TC5 TC6R TC7R TC8 TC9R TC10 TC11 TC12 TC13 TC14 GP1 GP2 GP3 GP4 GP5 GP6 GP7 GP8 GP9 GP10 GP11 GP12 SK1 SK2 SK3 SK4 SK5 SK6R SK7R SK8 SK9 SK10 SK11 SK12 EQ1 EQ2 EQ3 EQ4 EQ5R EQ6 EQ7 EQ8 EQ9 EQ10R EQ11R EQ12R EQ13 EQ14 ;

Missing are . ;

USEVARIABLES are

OV3 OV6

OV7 OV8

PH2 PH3 PH4

PH5R PH6R PH9R PH10R

CO2 CO3 CO4 CO6R

CO7R CO8R

TC11 TC12

TC13 TC14

GP4

GP7 GP8 GP10

SK5 SK8 SK12

EQ1 EQ2 EQ3 EQ4

;

ANALYSIS: ESTIMATOR=ML;

! ROTATION = VARIMAX;

MODEL: OV_READ BY OV3 OV6 OV7 OV8 ;

PHYSGOOD BY PH2 PH3 PH4 ;

PHYSBAD BY PH5R PH6R PH9R PH10R ;

COGGOOD BY CO2 CO3 CO4 ;

COG_BAD BY CO6R CO7R CO8R ;

THR_CHAL BY TC11 TC12 TC13 TC14 ;

GP_TEAM BY GP4 GP7 GP8 GP10 ;

SK_TRN BY SK5 SK8 SK12 ;

EQUIP BY EQ1 EQ2 EQ3 EQ4 ;

OV_READ ON PHYSGOOD PHYSBAD COGGOOD

COG_BAD THR_CHAL GP_TEAM SK_TRN

EQUIP ;

Or

SUM_READ BY OV_READ PHYSGOOD PHYSBAD COGGOOD

COG_BAD THR_CHAL GP_TEAM SK_TRN

EQUIP ;

OUTPUT: STDYX MODINDICES(ALL) ;
