# Supplementary material for: Development and Initial Validation of an Acute Readiness Monitoring Scale in Military Personnel
Source: Front Psychol. 2021 Nov 18;12:738609. doi: 10.3389/fpsyg.2021.738609 (PMC8636321; doi:10.3389/fpsyg.2021.738609)
Supplement: Supplementary file 3 [file Data_Sheet_2.pdf]

The questions in the ARMS:

Please answer the following questions in relation to how ready you feel for any upcoming task or challenge.

#	Factor	Item wording	**Response right now**<br>Does not apply at all ... Fully applies	Reverse code
1	Overall Readiness	I feel ready to deal with serious threats	0------------1------------2------------3------------4------------5-----------6	
2		My skills and experience make me capable of meeting any challenge	0------------1------------2------------3------------4------------5-----------6	
3		I feel ready to deal with uncertainty	0------------1------------2------------3------------4------------5-----------6	
4		I feel confident in taking control of situations	0------------1------------2------------3------------4------------5-----------6	
5	Physical Readiness	I am physically fit	0------------1------------2------------3------------4------------5-----------6	
6		I am physically prepared	0------------1------------2------------3------------4------------5-----------6	
7		I am physically fresh	0------------1------------2------------3------------4------------5-----------6	
8	Physical Fatigue	I am physically tired	0------------1------------2------------3------------4------------5-----------6	R
9		My muscles are sore	0------------1------------2------------3------------4------------5-----------6	R
10		I am fatigued	0------------1------------2------------3------------4------------5-----------6	R
11		I am physically spent	0------------1------------2------------3------------4------------5-----------6	R
12	Cognitive Readiness	I can focus well	0------------1------------2------------3------------4------------5-----------6	
13		I am mentally prepared	0------------1------------2------------3------------4------------5-----------6	
14		I am thinking clearly	0------------1------------2------------3------------4------------5-----------6	
15	Cognitive Fatigue	I am mentally tired	0------------1------------2------------3------------4------------5-----------6	R
16		My mind is fuzzy today	0------------1------------2------------3------------4------------5-----------6	R
17		I cannot focus today	0------------1------------2------------3------------4------------5-----------6	R
18	Threat-Challenge Readiness	I am ready to process significant problems	0------------1------------2------------3------------4------------5-----------6	
19		No matter the challenge, I am ready for it	0------------1------------2------------3------------4------------5-----------6	
20		I have things under control today	0------------1------------2------------3------------4------------5-----------6	
21		I can handle unpleasant feelings	0------------1------------2------------3------------4------------5-----------6	
22	Group-Team Readiness	My team is ready	0------------1------------2------------3------------4------------5-----------6	
23		My team has strong systems and processes	0------------1------------2------------3------------4------------5-----------6	
24		My team works well together	0------------1------------2------------3------------4------------5-----------6	
25		I have confidence in my team	0------------1------------2------------3------------4------------5-----------6	
26	Skills-Training Readiness	I offer significant value to my role/Unit	0------------1------------2------------3------------4------------5-----------6	
27		I am capable of delivering my role	0------------1------------2------------3------------4------------5-----------6	
28		I feel confident in my abilities to perform my role	0------------1------------2------------3------------4------------5-----------6	
29	Equipment Readiness	I have all the equipment I need	0------------1------------2------------3------------4------------5-----------6	
30		My equipment is well-maintained	0------------1------------2------------3------------4------------5-----------6	
31		My equipment is fit-for-purpose	0------------1------------2------------3------------4------------5-----------6	
32		My equipment is world-leading	0------------1------------2------------3------------4------------5-----------6	
