# Supplementary material for: Development and Initial Validation of an Acute Readiness Monitoring Scale in Military Personnel
Source: Front Psychol. 2021 Nov 18;12:738609. doi: 10.3389/fpsyg.2021.738609 (PMC8636321; doi:10.3389/fpsyg.2021.738609)
Supplement: Supplementary file 4 [file Table_1.DOCX]

**Section 1 - About your readiness**

Please answer the following questions in relation to how ready you feel for any upcoming task or challenge.

| **Total** | **Item** | **Question wording – OVERALL READINESS** | **Response right now** |
| --- | --- | --- | --- |
| 1 | OV1 | I feel physically ready for any challenge | Does not Fully  apply at all applies    0------------1------------2------------3------------4------------5-----------6 |
| 2 | OV2 | I feel mentally ready for any challenge | Does not Fully  apply at all applies    0------------1------------2------------3------------4------------5-----------6 |
| 3 | OV3 | I feel ready to deal with serious threats | Does not Fully  apply at all applies    0------------1------------2------------3------------4------------5-----------6 |
| 4 | OV4 | My team is ready to work together | Does not Fully  apply at all applies    0------------1------------2------------3------------4------------5-----------6 |
| 5 | OV5 | My equipment is fit-for-purpose | Does not Fully  apply at all applies    0------------1------------2------------3------------4------------5-----------6 |
| 6 | OV6 | My skills and experience make me capable of meeting any challenge | Does not Fully  apply at all applies    0------------1------------2------------3------------4------------5-----------6 |
| 7 | OV7 | I feel ready to deal with uncertainty | Does not Fully  apply at all applies    0------------1------------2------------3------------4------------5-----------6 |
| 8 | OV8 | I feel confident in taking control of situations | Does not Fully  apply at all applies    0------------1------------2------------3------------4------------5-----------6 |
| 9 | OV9 | I feel confident that my actions contribute to our overall objectives | Does not Fully  apply at all applies    0------------1------------2------------3------------4------------5-----------6 |
| 10 | OV10 | I feel ready for any physically demanding task | Does not Fully  apply at all applies    0------------1------------2------------3------------4------------5-----------6 |
| 11 | OV11 | I feel ready for any mentally demanding task | Does not Fully  apply at all applies    0------------1------------2------------3------------4------------5-----------6 |
| 12 | OV12 | I feel ready to deal with stressful situations | Does not Fully  apply at all applies    0------------1------------2------------3------------4------------5-----------6 |

| 13 | PH1 | I am physically strong | Does not Fully  apply at all applies    0------------1------------2------------3------------4------------5-----------6 |
| --- | --- | --- | --- |
| 14 | PH2 | I am physically fit | Does not Fully  apply at all applies    0------------1------------2------------3------------4------------5-----------6 |
| 15 | PH3 | I am physically prepared | Does not Fully  apply at all applies    0------------1------------2------------3------------4------------5-----------6 |
| 16 | PH4 | I am physically fresh | Does not Fully  apply at all applies    0------------1------------2------------3------------4------------5-----------6 |
| 17 | PH5 | I am physically tired | Does not Fully  apply at all applies    0------------1------------2------------3------------4------------5-----------6 |
| 18 | PH6 | My muscles are sore | Does not Fully  apply at all applies    0------------1------------2------------3------------4------------5-----------6 |
| 19 | PH7 | I feel energetic | Does not Fully  apply at all applies    0------------1------------2------------3------------4------------5-----------6 |
| 20 | PH8 | I am ready to exert physical effort | Does not Fully  apply at all applies    0------------1------------2------------3------------4------------5-----------6 |
| 21 | PH9 | I am fatigued | Does not Fully  apply at all applies    0------------1------------2------------3------------4------------5-----------6 |
| 22 | PH10 | I am physically spent | Does not Fully  apply at all applies    0------------1------------2------------3------------4------------5-----------6 |
| 23 | PH11 | I am sick today | Does not Fully  apply at all applies    0------------1------------2------------3------------4------------5-----------6 |
| 24 | PH12 | I am injured today | Does not Fully  apply at all applies    0------------1------------2------------3------------4------------5-----------6 |

| 25 | CO1 | I am mentally fit | Does not Fully  apply at all applies    0------------1------------2------------3------------4------------5-----------6 |
| --- | --- | --- | --- |
| 26 | CO2 | I can focus well | Does not Fully  apply at all applies    0------------1------------2------------3------------4------------5-----------6 |
| 27 | CO3 | I am mentally prepared | Does not Fully  apply at all applies    0------------1------------2------------3------------4------------5-----------6 |
| 28 | CO4 | I am thinking clearly | Does not Fully  apply at all applies    0------------1------------2------------3------------4------------5-----------6 |
| 29 | CO5 | I am mentally fresh | Does not Fully  apply at all applies    0------------1------------2------------3------------4------------5-----------6 |
| 30 | CO6 | I am mentally tired | Does not Fully  apply at all applies    0------------1------------2------------3------------4------------5-----------6 |
| 31 | CO7 | My mind is fuzzy today | Does not Fully  apply at all applies    0------------1------------2------------3------------4------------5-----------6 |
| 32 | CO8 | I cannot focus today | Does not Fully  apply at all applies    0------------1------------2------------3------------4------------5-----------6 |
| 33 | CO9 | I am ready to problem solve today | Does not Fully  apply at all applies    0------------1------------2------------3------------4------------5-----------6 |
| 34 | CO10 | I feel alert | Does not Fully  apply at all applies    0------------1------------2------------3------------4------------5-----------6 |
| 35 | CO11 | I am easily distracted today | Does not Fully  apply at all applies    0------------1------------2------------3------------4------------5-----------6 |
| 36 | CO12 | I have no willpower today | Does not Fully  apply at all applies    0------------1------------2------------3------------4------------5-----------6 |

| 37 | TC1 | I am in a good mood | Does not Fully  apply at all applies    0------------1------------2------------3------------4------------5-----------6 |
| --- | --- | --- | --- |
| 38 | TC2 | I feel in a good place today | Does not Fully  apply at all applies    0------------1------------2------------3------------4------------5-----------6 |
| 39 | TC3 | I feel threatened | Does not Fully  apply at all applies    0------------1------------2------------3------------4------------5-----------6 |
| 40 | TC4 | I feel anxious | Does not Fully  apply at all applies    0------------1------------2------------3------------4------------5-----------6 |
| 41 | TC5 | I can manage my emotions today | Does not Fully  apply at all applies    0------------1------------2------------3------------4------------5-----------6 |
| 42 | TC6 | I feel annoyed | Does not Fully  apply at all applies    0------------1------------2------------3------------4------------5-----------6 |
| 43 | TC7 | Nothing can upset me today | Does not Fully  apply at all applies    0------------1------------2------------3------------4------------5-----------6 |
| 44 | TC8 | I feel down today | Does not Fully  apply at all applies    0------------1------------2------------3------------4------------5-----------6 |
| 45 | TC9 | I feel exhausted | Does not Fully  apply at all applies    0------------1------------2------------3------------4------------5-----------6 |
| 46 | TC10 | I could handle anything today | Does not Fully  apply at all applies    0------------1------------2------------3------------4------------5-----------6 |
| 47 | TC11 | I am ready to process significant problems | Does not Fully  apply at all applies    0------------1------------2------------3------------4------------5-----------6 |
| 48 | TC12 | No matter the challenge, I am ready for it | Does not Fully  apply at all applies    0------------1------------2------------3------------4------------5-----------6 |
| 49 | TC13 | I have things under control today | Does not Fully  apply at all applies    0------------1------------2------------3------------4------------5-----------6 |
| 50 | TC14 | I can handle unpleasant feelings | Does not Fully  apply at all applies    0------------1------------2------------3------------4------------5-----------6 |

| 51 | GP1 | I can talk to my team about problems/challenges | Does not Fully  apply at all applies    0------------1------------2------------3------------4------------5-----------6 |
| --- | --- | --- | --- |
| 52 | GP2 | I feel supported by people around me | Does not Fully  apply at all applies    0------------1------------2------------3------------4------------5-----------6 |
| 53 | GP3 | My team is functioning well | Does not Fully  apply at all applies    0------------1------------2------------3------------4------------5-----------6 |
| 54 | GP4 | My team is ready | Does not Fully  apply at all applies    0------------1------------2------------3------------4------------5-----------6 |
| 55 | GP5 | My team is united | Does not Fully  apply at all applies    0------------1------------2------------3------------4------------5-----------6 |
| 56 | GP6 | My team has clear goals | Does not Fully  apply at all applies    0------------1------------2------------3------------4------------5-----------6 |
| 57 | GP7 | My team has strong systems and processes | Does not Fully  apply at all applies    0------------1------------2------------3------------4------------5-----------6 |
| 58 | GP8 | My team works well together | Does not Fully  apply at all applies    0------------1------------2------------3------------4------------5-----------6 |
| 59 | GP9 | My team can do more together than as individuals | Does not Fully  apply at all applies    0------------1------------2------------3------------4------------5-----------6 |
| 60 | GP10 | I have confidence in my team | Does not Fully  apply at all applies    0------------1------------2------------3------------4------------5-----------6 |
| 61 | GP11 | I have confidence in my team's leadership | Does not Fully  apply at all applies    0------------1------------2------------3------------4------------5-----------6 |
| 62 | GP12 | Our team is sufficiently trained and equipped to achieve operational effectiveness | Does not Fully  apply at all applies    0------------1------------2------------3------------4------------5-----------6 |

| 63 | SK1 | I have the skills and competencies required for my role | Does not Fully  apply at all applies    0------------1------------2------------3------------4------------5-----------6 |
| --- | --- | --- | --- |
| 64 | SK2 | I can adapt when things don't go well | Does not Fully  apply at all applies    0------------1------------2------------3------------4------------5-----------6 |
| 65 | SK3 | I can adapt to new problems | Does not Fully  apply at all applies    0------------1------------2------------3------------4------------5-----------6 |
| 66 | SK4 | I know my role | Does not Fully  apply at all applies    0------------1------------2------------3------------4------------5-----------6 |
| 67 | SK5 | I offer significant value to my role/Unit | Does not Fully  apply at all applies    0------------1------------2------------3------------4------------5-----------6 |
| 68 | SK6 | I question my abilities | Does not Fully  apply at all applies    0------------1------------2------------3------------4------------5-----------6 |
| 69 | SK7 | I feel under-prepared | Does not Fully  apply at all applies    0------------1------------2------------3------------4------------5-----------6 |
| 70 | SK8 | I am capable of delivering my role | Does not Fully  apply at all applies    0------------1------------2------------3------------4------------5-----------6 |
| 71 | SK9 | I feel able to meet the challenge of performing my role | Does not Fully  apply at all applies    0------------1------------2------------3------------4------------5-----------6 |
| 72 | SK10 | My training and experience have been the ideal preparation | Does not Fully  apply at all applies    0------------1------------2------------3------------4------------5-----------6 |
| 73 | SK11 | I am good at adapting to unfamiliar challenges | Does not Fully  apply at all applies    0------------1------------2------------3------------4------------5-----------6 |
| 74 | SK12 | I feel confident in my abilities to perform my role | Does not Fully  apply at all applies    0------------1------------2------------3------------4------------5-----------6 |

| 75 | EQ1 | I have all the equipment I need | Does not Fully  apply at all applies    0------------1------------2------------3------------4------------5-----------6 |
| --- | --- | --- | --- |
| 76 | EQ2 | My equipment is well-maintained | Does not Fully  apply at all applies    0------------1------------2------------3------------4------------5-----------6 |
| 78 | EQ3 | My equipment is fit-for-purpose | Does not Fully  apply at all applies    0------------1------------2------------3------------4------------5-----------6 |
| 79 | EQ4 | My equipment is world-leading | Does not Fully  apply at all applies    0------------1------------2------------3------------4------------5-----------6 |
| 80 | EQ5 | My equipment is faulty/broken | Does not Fully  apply at all applies    0------------1------------2------------3------------4------------5-----------6 |
| 81 | EQ6 | I have confidence in the equipment I use | Does not Fully  apply at all applies    0------------1------------2------------3------------4------------5-----------6 |
| 82 | EQ7 | I am confident our equipment will meet the challenges we face | Does not Fully  apply at all applies    0------------1------------2------------3------------4------------5-----------6 |
| 83 | EQ8 | My equipment makes me more capable | Does not Fully  apply at all applies    0------------1------------2------------3------------4------------5-----------6 |
| 84 | EQ9 | My equipment makes me more effective | Does not Fully  apply at all applies    0------------1------------2------------3------------4------------5-----------6 |
| 85 | EQ10 | My equipment 'gets in the way' sometimes | Does not Fully  apply at all applies    0------------1------------2------------3------------4------------5-----------6 |
| 86 | EQ11 | I am worried my equipment will fail | Does not Fully  apply at all applies    0------------1------------2------------3------------4------------5-----------6 |
| 87 | EQ12 | I am worried my equipment will let me down | Does not Fully  apply at all applies    0------------1------------2------------3------------4------------5-----------6 |
| 88 | EQ13 | I know how to maintain my equipment | Does not Fully  apply at all applies    0------------1------------2------------3------------4------------5-----------6 |
| 89 | EQ14 | I know how to service and repair my equipment | Does not Fully  apply at all applies    0------------1------------2------------3------------4------------5-----------6 |
